# Validation of Small‐Molecule Entry Inhibitors Targeting the Respiratory Syncytial Virus

**DOI:** 10.1002/cmdc.70501

**Published:** 2026-10-21

**Authors:** Christina Karhan, Svenja M. Sake, Melanie Urbanek‐Quaing, Diletta Bergamo, Jessica Rückert, Jiabin Huang, Christina Grethe, Antonia P. Gunesch, Anne Kühnel, Birthe Reinecke, Nicole Fischer, Thomas F. Schulz, Thomas Pietschmann, Anna K. H. Hirsch

**Affiliations:** ^1^ Helmholtz‐Institute for Pharmaceutical Research Saarland (HIPS) – Helmholtz Centre for Infection Research (HZI) Department of Pharmacy Saarland University Saarbrücken Germany; ^2^ TWINCORE, Centre for Experimental and Clinical Infection Research A Joint Venture Between the Helmholtz Centre for Infection Research and the Hannover Medical School Hannover Germany; ^3^ PharmaScienceHub (PSH) Saarland University Saarbrücken Germany; ^4^ Medizinische Hochschule Hannover‐Institut für Virologie, MHH Hannover Germany; ^5^ Institute of Medical Microbiology, Virology and Hygiene University Medical Center Hamburg‐Eppendorf Hamburg Germany; ^6^ German Centre for Infection Research Partner Site Hamburg‐Borstel‐Lübeck‐Riems Hamburg Germany; ^7^ Institute for Molecular Virology and Tumorvirology University Medical Center Hamburg Eppendorf Hamburg Germany; ^8^ German Center for Lung Research (DZL) Biomedical Research in Endstage and Obstructive Lung Disease Hannover, (BREATH) Hannover Germany; ^9^ Cluster of Excellence RESIST (EXC 2155) Hanover Medical School Hannover Germany

**Keywords:** high‐throughput screening, hit validation, respiratory syncytial virus (RSV) entry inhibitors, small molecules

## Abstract

Respiratory syncytial virus (RSV) is a major cause of severe respiratory infections worldwide, particularly in infants, young children, and elderly individuals. Despite the availability of preventive options such as vaccines and monoclonal antibodies, effective antiviral therapies for acute infections remain limited. Therefore, we investigated small‐molecule entry inhibitors identified in a high‐throughput campaing and evaluated the most promising candidates in detail by performing resynthesis and determining their antiviral activity. Unfortunately, analytical investigations by nuclear magnetic resonance (NMR) spectroscopy and liquid chromatography–mass spectrometry (LC–MS) revealed discrepancies between the screening samples and the resynthesized compounds for two compound classes, highlighting the complexity of hit validation and the potential influence of impurities. By publishing these results, we aim not only to underscore the importance of rigorous analytical and biological validation in screening campaigns but also to support the development of improved strategies for identifying reliable RSV inhibitors. We also believe it is important to publish false‐positive hits to avoid time efforts on projects that assume such compounds as genuine hits.

## Graphical abstract

Respiratory syncytial virus (RSV) remains a major cause of severe respiratory disease, while effective treatments for acute infection are still limited. We screened small‐molecule RSV entry inhibitors and investigated promising hits through resynthesis and antiviral testing. NMR and LC–MS analyses revealed discrepancies between screening samples and resynthesized compounds, emphasizing the potential influence of impurities. These findings highlight the critical importance of rigorous chemical and biological validation in drug discovery.

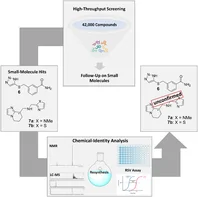

## 1 Introduction

The coronavirus pandemic has emphasized the importance of developing antiviral agents to safeguard human health, highlighting that the treatment of severe viral infections persisting despite vaccination continues to be a key goal in pharmaceutical science.

Besides corona viruses, numerous other pathogens can induce severe cases of viral infections such as the respiratory syncytial virus (RSV). Approximately 100,000 of around 3.6 million RSV‐associated hospitalizations worldwide per year are children under the age of five, half of whom die at less than 6 months of age. For adults and older people over 60, infection is fatal in around 10% of hospitalized patients [1]. RSV belongs to the *Pneumoviridae* family and is an enveloped virus. Its envelope is a typical biological membrane consisting of a lipid bilayer equipped with membrane proteins including the attachment (G) and the fusion (F) proteins [2]. Both the G protein and the F protein are involved in viral entry. While the G protein supports the attachment of the virion to the host cell and also modulates the host’s response to the infection [3], the F protein is responsible for the fusion of the virus with the host cell [4].

Although monoclonal antibodies such as palivizumab (Synagis) and nirsevimab (Beyfortus) [5, 6] targeting the RSV‐F protein have been extensively studied and licensed for prevention of RSV infection in infants, development of directly acting antivirals for treatment of RSV infections remains highly important. Among the small‐molecule antivirals against RSV, projects to date have mainly focused on the F, L, and N proteins [7, 8]. Only one small‐molecule inhibitor has addressed the G protein [9]. In contrast to the G protein, which is a highly variable structural protein, the F protein is highly conserved among RSV isolates with amino acid sequence identities of at least 90%. This enables a better coverage between the viral subtypes RSV/A and RSV/B [2, 10].

The optimized version of BMS‐433771 (**1**), rilematovir (**2**) [11], presatovir (**3**) [12], sisunatovir (**4**) [13], and ziresovir (**5**) [14] are examples of RSV entry inhibitors that reached clinical studies (Figure 1). They all have in vitro activities in the nanomolar or even picomolar range and are orally bioavailable [11, 12, 14, 15, 16, 17].

**FIGURE 1:**
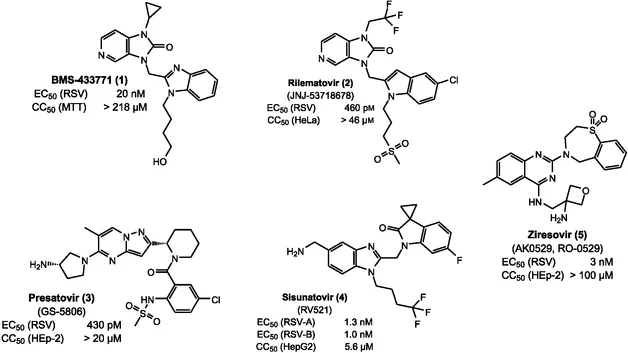
Overview of RSV entry inhibitors that reached clinical studies and corresponding half‐maximal effective (EC_50_) as well as cytotoxic (CC_50_) concentration values derived from literature [10, 12, 13, 14, 15].

Nowadays, there is no effective, FDA‐approved agent to treat severe RSV infections, although there are a few promising inhibitors in the pipeline. In order to supplement the portfolio of antiviral drugs available, to bring further backup inhibitors into the pipeline, and to be able to develop possible starting points for combination therapies, we embarked on a high‐throughput screening (HTS) campaing. In this screening, around 42,000 compounds including small molecules, drug repositioning candidates from the ReFrame library [18], and natural products were tested in a two‐step RSV reporter virus infection assay [19, 20]. Among the small molecules, ten hit classes with half‐maximal inhibitory concentrations (IC_50_) less than 10 µM were promising, wherefore we followed‐up on them. While four of these hit classes have already been published elsewhere [19], two hit classes (**6** and **7**, Figures 2 and S12A,B, S19A,B) have not been described yet. RSV passage in the presence of increasing amounts of the reordered screening hits **6** and **7** led to outgrowth of virus populations that were phenotypically resistant against their respective compounds (data not shown). The virus populations were sequenced and several nonsynonomous variants in the F‐protein coding sequence had accumulated, suggesting the F‐protein and virus entry as the putative molecular and mechanistic target (Figures S12C and S19C). Since the compound‐adapted virus populations were cross‐resistant to each other and to known RSV‐fusion inhibitors targeting the F protein (presatovir as well as BMS‐433771) (data not shown), we concluded that compounds **6** and **7** likely interact with the RSV‐F protein and thereby inhibit entry of the virus particle into the host cell. Overall, the ordered compounds **6** and **7** had been characterized as RSV entry inhibitors and belonged to the most active, noncytotoxic screening hits. Unfortunately, the observed antiviral effect could not be attributed to the specified molecular structures. Their hit‐confirmation process and chemical‐identity analysis are discussed below.

**FIGURE 2:**
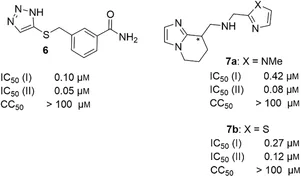
Two hit classes from high‐throughput screening (HTS). Corresponding half‐maximal concentrations (IC_50_) and cytotoxic concentrations (CC_50_) determined in the first (I) and second (II) infection rounds of the RSV screen are displayed.

## 2 Results and Discussion

### 2.1 Compound Class 6

To confirm the chemical identity of screening hit **6**, we performed the compound’s resynthesis to obtain a reference compound (**6–‍1**, Scheme 1) and analyzed the screening sample via liquid chromatography–mass spectrometry (LC–MS) and nuclear magnetic resonance (NMR) spectroscopy. The resynthesis consists of two steps, starting with a nucleophilic substitution that resulted in intermediate **10**. This intermediate was coupled to ammonia, affording the primary amide **6–1**.

**SCHEME 1:**
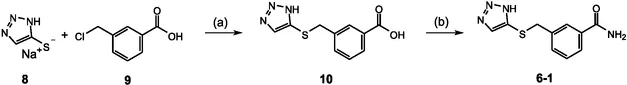
Resynthesis of screening hit **6**—reagents and conditions. (a) Ethanol (EtOH), reflux, 4 h, 32% and (b) ammonia (0.5 M in 1,4‐dioxane), NEt_3_, 2‐(1*H*‐benzotriazole‐1‐yl)‐1,1,3,3‐tetramethylaminium tetrafluoroborate (TBTU), DMF, closed vessel (c. v), r. t., 3 h, 29%.

Unfortunately, the LC–MS chromatogram of the initial screening hit **6** showed only a purity of 62% (UV‐peak integration of UV chromatogram, Figure S1). The mass of the first peak does not belong to compound **6** or **6–1**, respectively. Instead, it fits to the mass of intermediate **10**, but not to its retention time when compared to LC–MS data of synthesized intermediate **10** (Figure S2). Nevertheless, we tested intermediate **10** in a two‐step RSV infection assay in cell culture [12]. As expected, it was completely inactive against RSV infection (Table 1). Additionally, we compared the ^1^H NMR spectra of intermediate **10** and the resynthesized compound **6–1** to those of the screening sample (Figure S4). However, as already assumed on the basis of the LC–MS analysis, the overlay of ^1^H NMR spectra proved that neither the resynthesized compound **6–1** nor intermediate **10** was present in the screening sample. Therefore, we did not test the resynthesized compound **6–1** in the RSV infection assay.

**TABLE 1:** Activity against respiratory syncytial virus (RSV) and cell‐viability profiles of intermediate **10** compared to initial screening hit **6.** a

Compound		RSV		HEp‐2
		I	II	
		IC_50_, µM	IC_50_, µM	CC_50_, µM
		% Inf	% Inf	% CV
**6**	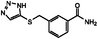	0.11	0.05	>100
		4 ± 1	3 ± 1^B^	103 ± 6
**10**	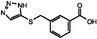	n.d.	n.d.	n.d.
		104 ± 9	67 ± 9^B^	102 ± 10

a Infectivity (Inf) and cell viability (CV) as well as half‐maximal inhibitory (IC_50_) and cytotoxic concentrations (CC_50_) of the tested compounds are listed. For the RSV‐infection assay, the cells were infected in two rounds, whereby virus containing supernatant from infection round 1 (I) was used to infect cells in round 2 (II) to get an indication of whether antiviral effects are due to effects against early (e.g., virus entry) or late (e.g., replication, budding) stages of the viral cycle. IC_50_ and CC_50_ determination are based on dose–response curves, while %inf and %CV determination are based on single‐point measurements performed in triplicate (counts (*n*) = 3) or duplicate (B) at 10 µM compound concentration. Mean values and corresponding standard deviations (SD) of these single‐point measurements are given. Dose‐response analyses were done once, if not described differently and only for compounds showing maximum 25% Inf in the first infection round; for their counts, the same legend as for the single‐point measurements applies. Additionally, the following abbreviation is used: n.d., not determined.

### 2.2 Compound Class 7

By analogy, to confirm the chemical identity of initial hit class **7**, we focused our analyses via LC–MS and ^1^H NMR spectroscopy on scaffold **7a** and compared these results later with those of the resynthesized compound.

LC–MS measurements did not lead to a concrete result, there was no clear UV activity, and the compound showed up with the injection peak (Figure S13). However, two masses were found—the one of the protonated, expected hit compound **7a** (*m/z* ([M + H]^+^) = 246.03, Figure S13) and a mass that could not be assigned (*m/z* = 258.02, Figure S13).

To obtain a reference compound, we resynthesized initial hit **7a** in a one‐step synthesis based on a reductive amination similar to a literature‐known procedure [21], as shown in Scheme 2.

**SCHEME 2:**
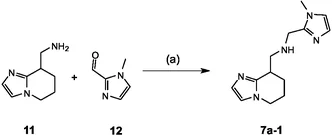
Resynthesis of screening hit **7a**—reagents and conditions. (a) 1) Acetic acid (AcOH), MeOH, r. t., 1 h; 2) sodium borohydride (NaBH_4_), r. t., 18 h, 19%.

Testing the resynthesized compound **7a–1** in the RSV‐infection assay, unfortunately did not lead to the desired result—it was inactive (Table 2).

**TABLE 2:** Activity against respiratory syncytial virus (RSV) and cell‐viability profiles of resynthesized compound **7a–1** compared to initial screening hits **7a** and **7b**.^a^

Compound		RSV		HEp‐2
		I	II	
		IC_50_, µM	IC_50_, µM	CC_50_, µM
**7a**	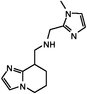	0.42	0.08	>100
**7b**	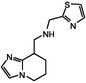	0.27	0.12	>100
**7a−1**	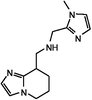	>3	n. d.	>100


a Half‐maximal inhibitory (IC_50_) and cytotoxic concentrations (CC_50_) of the tested compounds are listed. For the RSV infection assay, the cells were infected in only one round since the initial hit was classified as an entry inhibitor. Dose‐response analyses were done once if not described differently.

Comparing the ^1^H NMR spectra (Figure S15) and LC–MS data of the screening sample to those of the resynthesized compound revealed that **7a–1** is one of at least two components in this sample.

## 3 Conclusions

Analyses of compound classes **6** and **7** illustrate both the opportunities and challenges in early‐stage RSV inhibitor discovery. For class **6**, resynthesis and analytical studies revealed inconsistencies between the original screening sample and the synthesized candidates. The resynthesized hit compound **6–1** was not present in the screening sample. Therefore, it was not tested against RSV. Instead, we identified the mass of the close derivative **10** as the major fraction of the compound mixture, even though it showed a different retention time and proved to be inactive. In future studies, either fractionation of the screening sample by semiprep HPLC in combination with analysis of each fraction by NMR spectroscopy and additional biological testing should be performed. Similarly, for class **7**, resynthesis failed to reproduce the antiviral activity of the initial screening hits, and we could not confirm the chemical identity of the active species. These findings emphasize the complexity of hit validation in HTS campaigns, where impurities or unidentified components may account for apparent activity. Nevertheless, the investigations provided valuable methodological insights, emphasizing the need for rigorous hit confirmation.

## 4 Experimental Section

### 4.1 General Information

All chemicals were used as obtained from commercial suppliers without further purification. ^1^H and ^13^C NMR spectra were recorded on a Bruker Fourier 500 [500 MHz (^1^H), 126 MHz (^13^C)] spectrometer. Chemical shifts are given in parts per million (ppm) and referenced against the residual dimethyl sulfoxide‐*d*_6_ (DMSO‐*d*_6_) peak, while coupling constants (*J*) are indicated in hertz (Hz). Multiplicities are described with singlet (s), broad singlet (br s), doublet (d), doublet of a doublet (dd), doublet of a doublet of a doublet (ddd), triplet (t), doublet of a triplet (dt), quartet (q), doublet of a quartet (dq), sextet (sxt), septet (sept), and multiplet (m).

For reaction controls, thin‐layer chromatography (TLC) and/or LC–MS were used. Low‐resolution mass analytics and purity control of final compounds were performed by applying a SpectraSystems‐MSQ LC–MS system (Thermo Fisher Scientific) consisting of pump, Hypersil Gold column (100 mm ×  2.1 mm, particle size 3 µm), autosampler, VWD detector, and an ESI quadrupole mass spectrometer. For LC–MS measurements, the following method was used: positive/negative mode, acetonitrile (MeCN) + 0.1% formic acid (FA)/water (H_2_O) + 0.1% FA, 5%—100% MeCN + 0.1% FA over 5.8 min, 0.7 mL/min, and wavelength (*λ*) = 254 nm.

For column chromatography (CC), either the automated flash column chromatography (AFC) system CombiFlash Rf 150 (Teledyne Isco) equipped with RediSepRf silica columns was used or manual flash column chromatography (MFC) with Silica 60 M, 0.04–0.063 mm or 0.063–0.2 mm (Macherey Nagel) was performed.

Final products were dried under high vacuum. In case the final compound was purified with semipreparative high‐performance liquid chromatography (semiprep HPLC), the corresponding isolated fraction was lyophilized using a Christ Alpha 2‐4 LD plus freeze‐dryer connected to Chemistry Hybrid Pump RC6 (Vacuubrand). For semiprep HPLC, an Ultimate 3000 ultra‐HPLC (UHPLC) system (Thermo Fisher Scientific) equipped with Dionex RS Pump, Diode Array Detector, Automated Fraction Collector, and Nucleodur C18 Gravity column (250 mm  ×  10 mm (column A) or 16 mm (column B), particle size 5 µM) was used [19]. All final and tested compounds were at least 95% pure by HPLC.

High‐resolution mass spectrometry (HRMS) measurements were conducted with a Q Exactive Focus (Thermo Fischer Scientific) connected to Dionex Ultimate 3000 RS Pump and Autosampler as well as UHPLC system Column compartment with Nucleodur C18 Pyramid column (150 mm × 2 mm, particle size 3 µm) and Diode Array Detector.

Characterizations of some compounds have partly already been published in different journals but sometimes resulted from other procedures. If not described differently, reactions have not been optimized [22].

Compounds were initially discovered in a small‐molecule screen involving the hRSV B05 eGFP reporter virus [23] (kindly provided by W. Paul Duprex of the University of Pittsburgh School of Medicine) at an MOI of 1 in the presence of 10 mM compound (1% DMSO) and using a Biomek FXP automation workstation for quantification of infection. Dose‐response validation of library small molecules, as shown in Figure S12, was conducted using an RSV‐A luciferase reporter virus as described recently [20].

Selection of virus resistance was performed as previously reported [20]. To further characterize these resistant virus populations and to identify F protein resistance mutations at the genetic level, the resistant viruses were subjected to deep sequencing. For this purpose, RSV RNA was purified from cell culture supernatants according to the manufacturer’s instructions. RNA Illumina NGS libraries were prepared from each sample after rRNA removal using the NEBNext rRNA Depletion Kit v2 and NEB Ultra II RNA library preparation. Libraries were multiplex‐sequenced on an Illumina MiSeq, generating about two million paired‐end reads per sample. Adapter sequences and low‐quality bases were trimmed with Trimmomatic v0.36 [24], and human sequences were filtered out using Bowtie2 v2.4.4 [25]. Remaining reads were aligned to the RSV‐GFP reference genome (GenBank accession no. MK816924) with Novoalign v3.07.00 (http://www.novocraft.com), duplicates were removed using Picard (http://broadinstitute.github.io/picard/), and intrahost single nucleotide variants (iSNVs) were identified with V‐Phaser2 [26], considering only variants supported by at least five reads per strand and with a strand‐bias ratio <10 between the two strands.

### 4.2 Compound Class 6

#### 4.2.1 3*‐*(((*1*H*‐1*,*2*,*3‐Triazol‐5‐yl*)*thio*)*methyl*)*benzoic acid* (*10*)

Thiolate **8** (72.1 mg, 0.59 mmol) and 3‐(chloromethyl)benzoic acid **9** (100.0 mg, 0.59 mmol, 1.0 equiv.) were heated to reflux in ethanol (EtOH, 5.9 mL, 0.1 M) for 4 h. After removing EtOH in vacuo, the resulting crude was acidified to pH 1–2 using aqueous HCl solution (1.0 M) purified by AFC (DCM/MeOH, 0%–10% MeOH over 20 min, 13 mL/min, 12 g silica, **10** at 5% MeOH), giving pure intermediate **10** (445.7 mg, 1.89 mmol, 32%) as a colorless solid. **R**_**f**_ (*v*/*v* (DCM/MeOH) = 97:3) = 0.12. **LC–MS** *m/z* ([M + H]^+^) = 236.00; *t*_r_ = 2.52 min; *purity*: >98%. ^**1**^ **H NMR** (500 MHz, DMSO‐*d*_6_) *δ*  =  15.13 (br s, 1H), 12.94 (br s, 1H), 7.84 (s, 1H), 7.79 (d, *J* = 7.8 Hz, 2H), 7.48 (br d, *J* = 7.5 Hz, 1H), 7.39 (t, *J* = 7.8 Hz, 1H), 4.22 (s, 2H). ^**13**^ **C NMR** (126 MHz, DMSO‐*d*_6_) *δ* = 167.1, 138.5, 133.3, 130.9, 129.7, 128.6, 128.1, 37.4 (one overlapping carbon signal at 128.6 (see HSQC spectrum (Figure S7), additionally two of the quaternary signals are overlapping, according to signal intensity, this could be the one at 130.9 ppm).

#### 4.2.2 3*‐*(((*1*H*‐1*,*2*,*3‐Triazol‐5‐yl*)*sulfinyl*)*methyl*)*benzoic acid* (*6–1*)

Intermediate **10** (58.0 mg, 0.25 mmol), NEt_3_ (38 µL, 0.27 mmol, 1.1 equiv.), and 2‐(1*H*‐benzotriazole‐1‐yl)‐1,1,3,3‐tetramethylaminium tetrafluoroborate (TBTU, 95.0 mg, 0.30 mmol, 1.2 equiv.) were stirred in DMF (1.0 mL, 0.25 M) at r. t. for 10 min before ammonia (0.5 M in 1,4‐dioxane) was added. The resulting reaction mixture was stirred for 3 h in a closed vessel (c. v.). Then, the solvent was removed in vacuo, the residue was dissolved in EtOAc and washed with aqueous KHSO_4_ solution (1.0 M) and saturated, aqueous NaCl solution. The organic layer was dried over Na_2_SO_4_, filtered, and concentrated in vacuo before the crude was purified by MFC (*v*/*v* (DCM/MeOH) = 97:3) and additional semiprep HPLC (H_2_O (+0.05% FA)/MeCN (+0.05% FA), 5%–100% MeCN, column A, 5 mL/min, **10** at 34% MeCN) giving **6–1** (16.9 mg, 0.07 mmol, 29%) as a colorless solid. **R**_**f**_ (*v*/*v* (DCM/MeOH) = 97:3) = 0.14. **LC–MS** *m/z* ([M + H]^+^) = 234.97; *t*_r_ = 2.03 min; *purity*: >98%. ^**1**^ **H NMR** (500 MHz, DMSO‐*d*_6_) *δ* = 15.17 (br s, 1H), 7.96 (br s, 1H), 7.83 (br s, 1H), 7.79 (s, 1H), 7.72 (d, *J* = 7.5 Hz, 1H), 7.39 (d, *J* = 7.5 Hz, 2H), 7.35 (t, *J* = 7.5 Hz, 1H), 4.20 (s, 2H). ^**13**^ **C NMR** (126 MHz, DMSO‐*d*_6_) *δ* = 167.6, 138.0, 134.4, 131.6, 128.3, 128.2, 126.2, 37.7 (one overlapping carbon signal at 131.6 (see HSQC spectrum (Figure S10)), additionally two of the quaternary signals are overlapping, according to signal intensity, this could be the one at 138.0 ppm). **HRMS** *calculated: m/z* ([M + H]^+^) = 235.0648, *found: m/z* ([M + H]^+^) = 235.0637.

### 4.3 Compound Class 7

#### 4.3.1 1*‐*(*1‐Methyl‐1*H*‐imidazol‐2‐yl*)*‐*N*‐*((*5*,*6*,*7*,*8‐tetrahydroimidazo*[*1*,*2‐a*]*pyridin‐8‐yl*)*methyl*)*methanamine* (*7a–1*)

For the synthesis of **7–1**, we followed a literature‐known procedure [21] and stirred amine **11** (20.0 mg, 0.13 mmol), commercial aldehyde **12** (16.5 mg, 0.15 mmol, 1.1 equiv.), and acetic acid (AcOH, 11 µL, 0.20 mmol, 1.5 equiv.) at r. t. in MeOH (1.0 mL, 0.13 M) for 1 h. Then, NaBH_4_ (25.0 mg, 0.66 mmol, 5.0 equiv.) was added, and the mixture was stirred at r.t. for further 18 h. After filtration using a nylon syringe filter, purification by preparative HPLC, and evaporation under vacuum at 40 °C in a in a Genevac, the desired product **7–1** (7 mg, 0.03 mmol, 19%) was obtained. **LC–MS** showed no UV activity; therefore, no purity analysis is possible. ^**1**^ **H NMR** (500 MHz, DMSO‐*d*_6_) *δ * =  7.03 (d, *J* = 0.9 Hz, 1H), 6.96 (d, *J* = 0.9 Hz, 1H), 6.79 (d, *J* = 1.1 Hz, 1H), 6.72 (d, *J* = 1.1 Hz, 1H), 3.96 (td, *J* = 4.7, 12.2 Hz, 1H), 3.85–3.77 (m, 1H), 3.74 (s, 2H), 3.64 (s, 3H), 2.89–2.78 (m, 2H), 2.70 (br s, 1H), 2.66–2.57 (m, 1H), 2.00–1.91 (m, 2H), 1.80 (tdd, *J* = 5.4, 10.8, 16.1 Hz, 1H), 1.59–1.48 (m, 1H). ^**13**^ **C NMR** (126 MHz, DMSO‐*d*_6_) *δ* =  146.6, 146.4, 126.8, 125.9, 121.5, 118.3, 52.8, 45.2, 44.2, 34.8, 32.3, 25.3, 21.5. **HRMS** *calculated m/z* ([M + H]^+^) = 246.1713, and *found m/z* ([M + H]^+^) = 246.1709.

## Funding

This study was supported by the VW‐Stiftung (9B812).

## Conflicts of Interest

The authors declare no conflicts of interest.

## Supporting information

Supplementary Material

## Data Availability

The data that supports the findings of this study are available in the Supporting Information of this article.

## References

[1] J. S. Nguyen‐Van‐Tam , M. O’Leary , E. T. Martin , et al., “Burden of Respiratory Syncytial Virus Infection in Older and High‐Risk Adults: A Systematic Review and Meta‐Analysis of the Evidence From Developed Countries,” European Respiratory Review 31, no. 166 (2022): 220105, 10.1183/16000617.0105-2022. PMC9724807 36384703

[2] M. B. Battles and J. S. McLellan , “Respiratory Syncytial Virus Entry and How to Block It,” Nature Reviews Microbiology 17, no. 4 (Apr. 2019): 233–245, 10.1038/s41579-019-0149-x. PMC7096974 30723301

[3] L. J. Anderson , S. J. Jadhao , C. R. Paden , and S. Tong , “Functional Features of the Respiratory Syncytial Virus G Protein,” Viruses 13, no. 7 (Jul 2021): 1214, 10.3390/v13071214. PMC8310105 34372490

[4] J. S. McLellan , W. C. Ray , and M. E. Peeples , “Structure and Function of Respiratory Syncytial Virus Surface Glycoproteins,” in Challenges and Opportunities for Respiratory Syncytial Virus Vaccines, ed. L. J. Anderson and B. S. Graham (Springer, 2013), 83–104, 10.1007/978-3-642-38919-1_4. PMC4211642 24362685

[5] O. of the Commissioner , “FDA Approves New Drug to Prevent RSV in Babies and Toddlers,” FDA., https://www.fda.gov/news‐e‍vents/ press‐announcements/fda‐approves‐new‐drug‐prevent‐rsv‐babies‐and‐toddlers.

[6] B. Resch , “Product Review on the Monoclonal Antibody Palivizumab for Prevention of Respiratory Syncytial Virus Infection, Human Vaccines & Immunotherapeutics 13, no. 9 (2017): 2138–2149, 10.1080/21645515.2017.1337614. PMC5612471 28605249

[7] G. S. Cockerill , J. A. D. Good , and N. Mathews , “State of the Art in Respiratory Syncytial Virus Drug Discovery and Development,” Journal of Medicinal Chemistry 62, no. 7 (2019): 3206–3227, 10.1021/acs.jmedchem.8b01361. 30411898

[8] G. Zou , S. Cao , Z. Gao , J. Yie , and J. Z. Wu , “Current State and Challenges in Respiratory Syncytial Virus Drug Discovery and Development,” Antiviral Research 221 (2024): 105791, 10.1016/j.antiviral.2023.105791. 38160942

[9] L. D. Saravolatz , K. M. Empey , R. S. Peebles Jr , and J. K. Kolls , “Pharmacologic Advances in the Treatment and Prevention of Respiratory Syncytial Virus,” Clinical Infectious Diseases 50, no. 9 (2010): 1258–1267, https://academic.oup.com/cid/article/50/9/1258/315638. 10.1086/651603 PMC2851489 20235830

[10] A. M. Hause , D. M. Henke , V. Avadhanula , C. A. Shaw , L. I. Tapia , and P. A. Piedra , “Sequence Variability of the Respiratory Syncytial Virus (RSV) Fusion Gene Among Contemporary and Historical Genotypes of RSV/a and RSV/B,” PLOS ONE 12, no. 4 (Apr. 2017): e0175792, 10.1371/journal.pone.0175792. PMC5393888 28414749

[11] D. Roymans , Alnajjar S. S. , Battles M. B. , et al., “Therapeutic Efficacy of a Respiratory Syncytial Virus Fusion Inhibitor,” Nature communications 8, no. 1 (2017): 167, 10.1038/s41467-017-00170-x. PMC5537225 28761099

[12] R. L. Mackman , M. Sangi , D. Sperandio , et al., “Discovery of an Oral Respiratory Syncytial Virus (RSV) Fusion Inhibitor (GS‐5806) and Clinical Proof of Concept in a Human RSV Challenge Study,” Journal of Medicinal Chemistry 58, no. 4 (2015): 1630–1643, https://pubs.acs.org/doi/full/10.1021/jm5017768. 10.1021/jm5017768 25574686

[13] G. S. Cockerill , R. M. Angell , A. Bedernjak , et al., “Discovery of Sisunatovir (RV521), an Inhibitor of Respiratory Syncytial Virus Fusion,” Journal of Medicinal Chemistry 64, no. 7 (2021): 3658–3676, https://pubs.acs.org/doi/full/10.1021/acs.jmedchem.0c01882. 10.1021/acs.jmedchem.0c01882 33729773

[14] X. Zheng , L. Gao , L. Wang , et al., “Discovery of Ziresovir as a Potent, Selective, and Orally Bioavailable Respiratory Syncytial Virus Fusion Protein Inhibitor,” Journal of Medicinal Chemistry 62, no. 13 (2019): 6003–6014, 10.1021/acs.jmedchem.9b00654. 31194544

[15] C. Cianci , K.‐L. Yu , K. Combrink , et al., “Orally Active Fusion Inhibitor of Respiratory Syncytial Virus,“ Antimicrobial Agents and Chemotherapy, 48, no. 2 (2004): 413–422, https://journals.asm.org/doi/full/10.1128/aac.48.2.413‐422.2004. 10.1128/AAC.48.2.413-422.2004 PMC321540 14742189

[16] PubChem “Rilematovir,” Accessed: Nov. 27, 2025, https://pubchem.ncbi.nlm.nih.gov/compound/118892432.

[17] PubChem , “Sisunatovir,” Accessed: Nov. 27, 2025, https://pubchem.ncbi.nlm.nih.gov/compound/132016492.

[18] “About ReframeDB,“ Accessed: Nov. 27, 2025, https://reframedb.org/about.

[19] S. M. Sake , C. Kosch , S. Blockus , et al., “Respiratory Syncytial Virus Two‐Step Infection Screen Reveals Inhibitors of Early and Late Life Cycle Stages,” Antimicrobial Agents and Chemotherapy 66, no. 12 (2022): e01032‐22, https://journals.asm.org/doi/full/10.1128/aac.01032‐22. 10.1128/aac.01032-22 PMC9765014 36346232

[20] S. M. Sake , X. Zhang , M. K. Rajak , et al., “Drug Repurposing Screen Identifies Lonafarnib as Respiratory Syncytial Virus Fusion Protein Inhibitor,” Nature Communications 15, no. 1 (2024): 1173, 10.1038/s41467-024-45241-y. PMC10853176 38332002

[21] M. A. Gil , W. Maringgele , S. Dechert , and F. Meyer , “Structural Flexibility of Carboxylate Bridging Exemplified by a Series of μ‐Acetato Dizinc Complexes,” Zeitschrift für Anorganische Und Allgemeine Chemie 633, no. 13‐14 (2007): 2178–2186, 10.1002/zaac.200700200.

[22] C. Karhan , S. M. Sake , A. P. Gunesch , et al., “Unlocking the Antiviral Arsenal: Structure‐Guided Optimization of Small‐Molecule Inhibitors Against RSV and hCoV‐229E,” European Journal of Medicinal Chemistry 291 (2025): 117282, 10.1016/j.ejmech.2025.117282. 40199027

[23] K. Lemon , D. T. Nguyen , M. Ludlow , et al., “Recombinant Subgroup B Human Respiratory Syncytial Virus Expressing Enhanced Green Fluorescent Protein Efficiently Replicates in Primary Human Cells and Is Virulent in Cotton Rats,” Journal of Virology 291 (2025): 117282, https://journals.asm.org/doi/full/10.1128/jvi.03587‐14. 10.1128/JVI.03587-14 PMC4325752 25540371

[24] A. M. Bolger , M. Lohse , and B. Usadel , “Trimmomatic: A Flexible Trimmer for Illumina Sequence Data,” Bioinformatics 30, no. 15 (2014): 2114–2120, 10.1093/bioinformatics/btu170. PMC4103590 24695404

[25] B. Langmead and S. L. Salzberg , “Fast Gapped‐Read Alignment With Bowtie 2,” Nature Methods 9, no. 4 (Apr. 2012): 357–359, 10.1038/nmeth.1923. PMC3322381 22388286

[26] X. Yang , P. Charlebois , A. Macalalad , M. R. Henn , and M. C. Zody , “V‐Phaser 2: Variant Inference for Viral Populations,” BMC Genomics 14, no. 1 (2013): 674, 10.1186/1471-2164-14-674. PMC3907024 24088188

