# Supplementary material for: Validation of Small‐Molecule Entry Inhibitors Targeting the Respiratory Syncytial Virus
Source: ChemMedChem. 2026 Oct 21;21(20):e70501. doi: 10.1002/cmdc.70501 (PMC13687780; doi:10.1002/cmdc.70501)
Supplement: Supplementary file 1 [file CMDC-21-e70501-s001.pdf]

Supporting information

Compound class 6

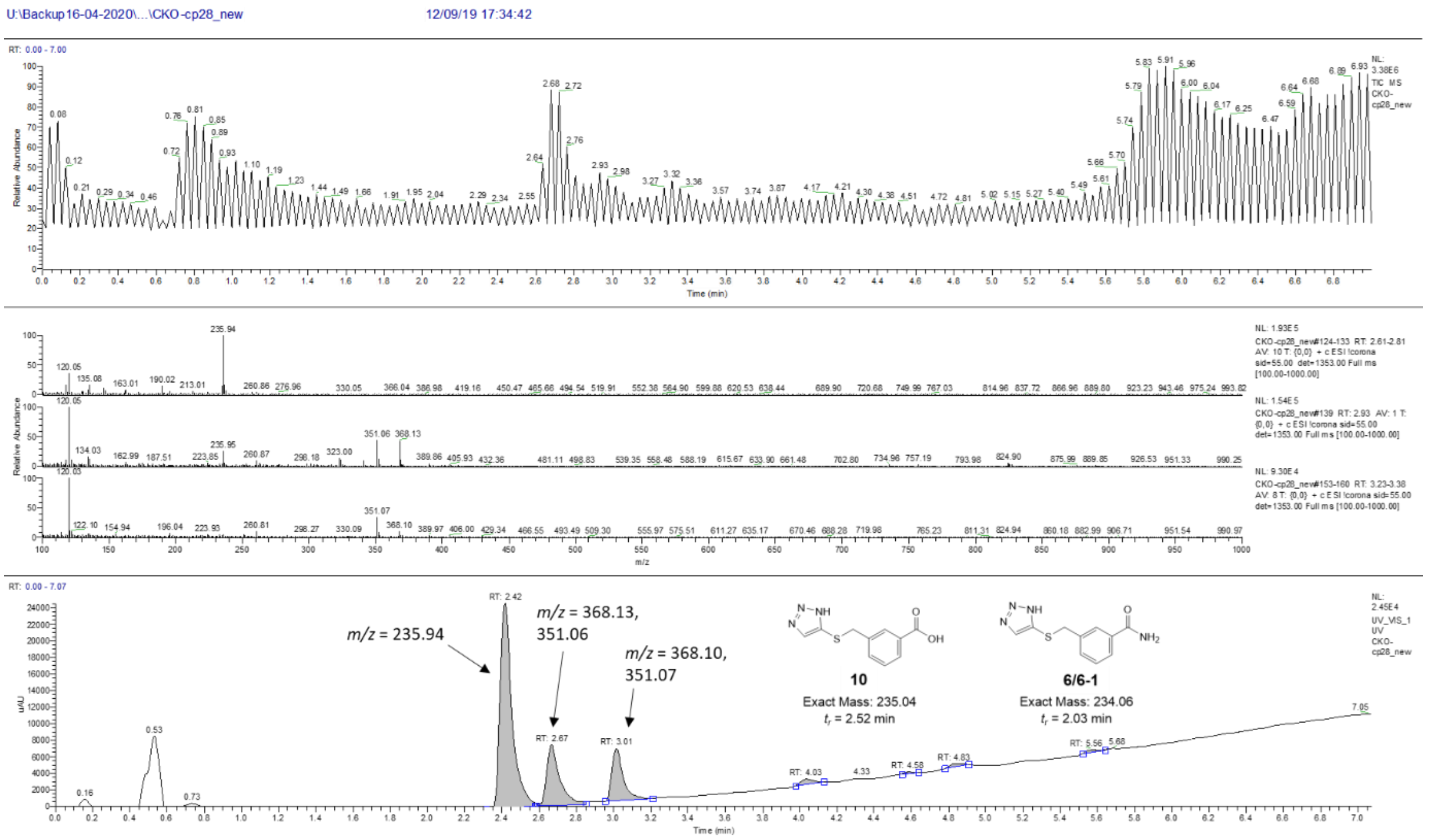

Figure S1: LC-MS data of initial screening sample (compound 6); top: mass chromatogram, middle: mass spectra of the three UV peaks, bottom: UV chromatogram.

LC-MS data

*3-(((1*H*-1,2,3-Triazol-5-yl)thio)methyl)benzoic acid (10)*

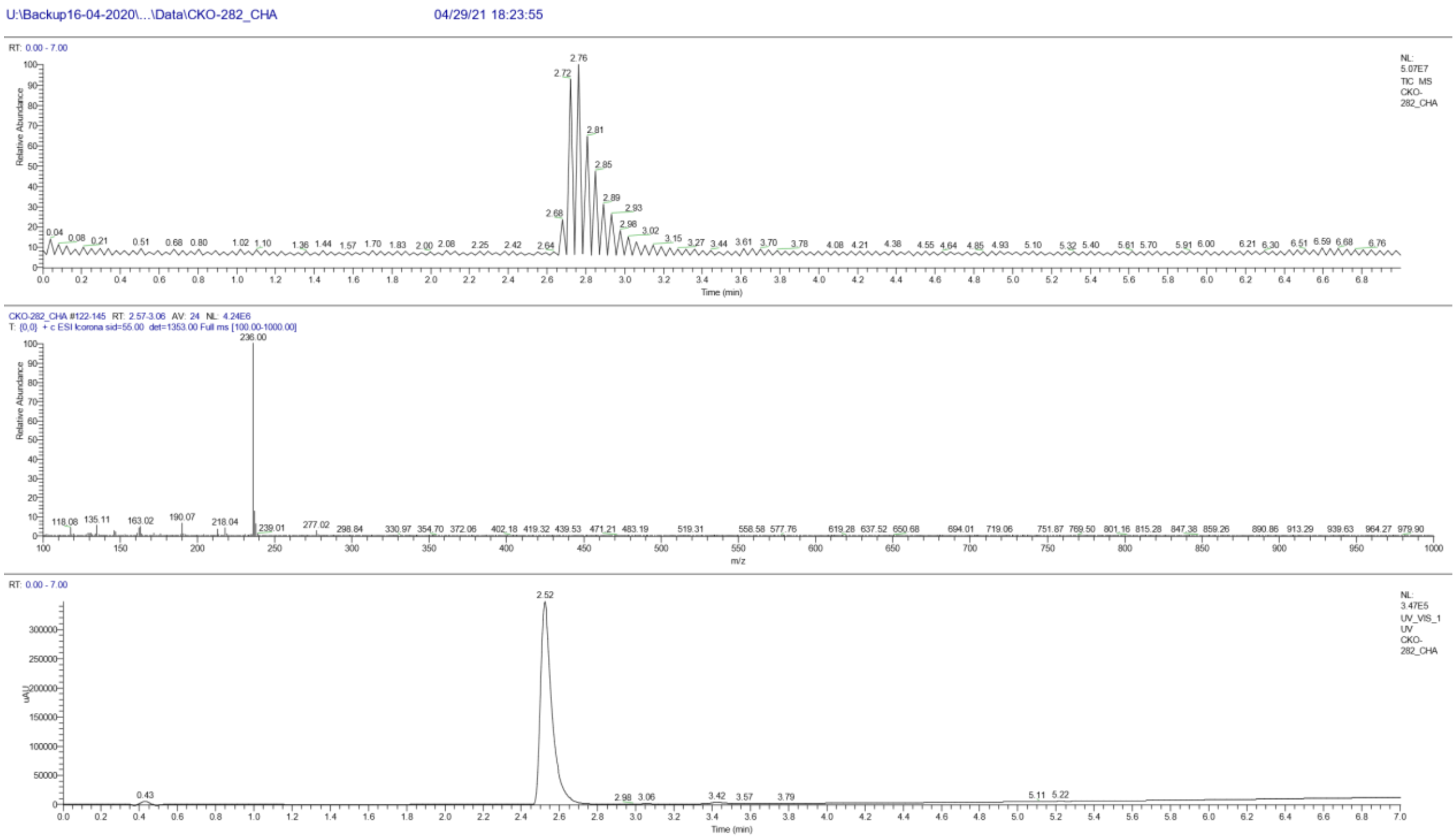

Figure S2: LC-MS data of **10** (top: mass chromatogram, middle: mass spectrum of UV/Vis peak, bottom: UV/Vis chromatogram).

3-(((1H-1,2,3-Triazol-5-yl)sulfinyl)methyl)benzoic acid (6-1)

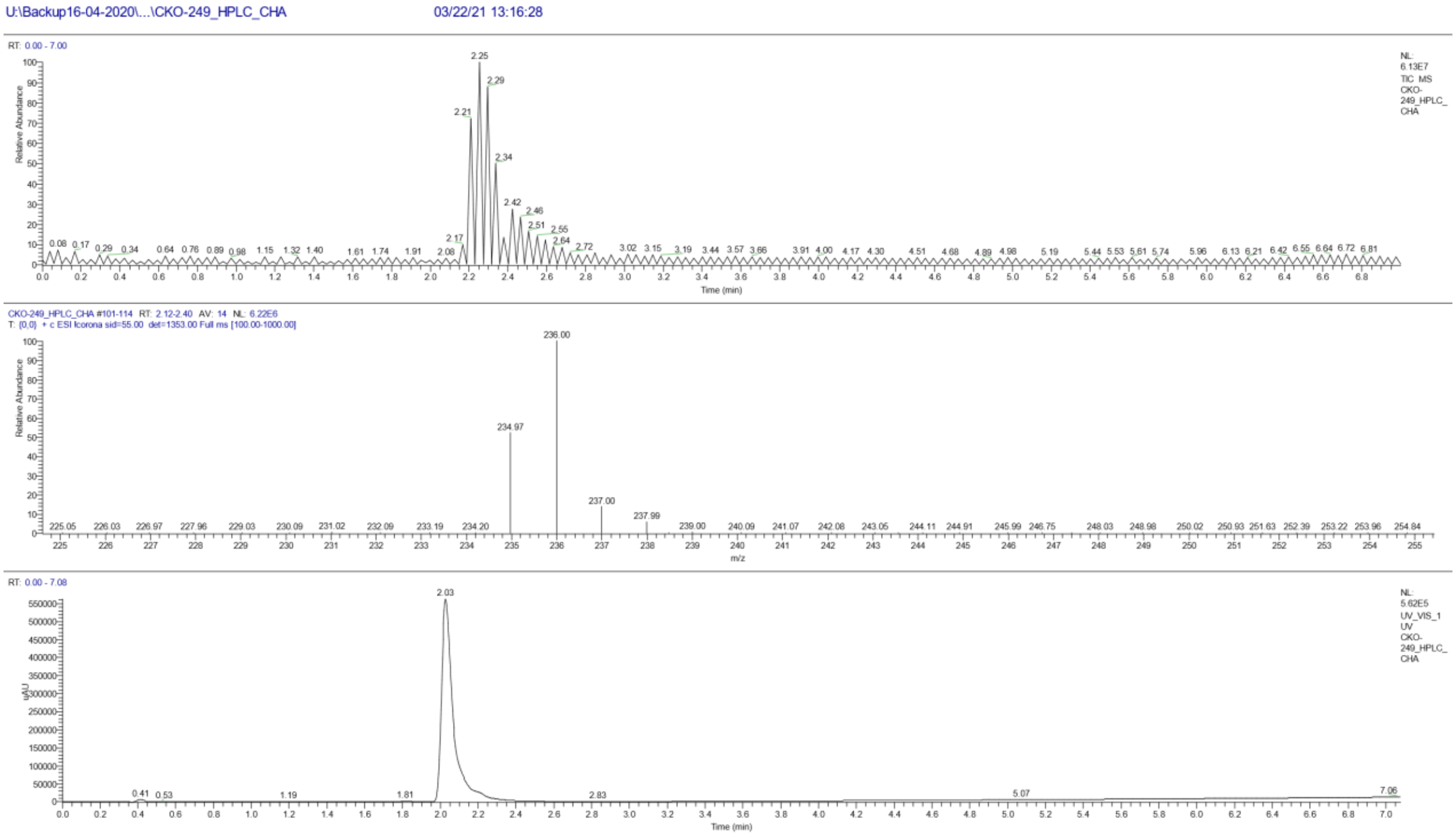

Figure S3: LC-MS data of **6-1** (top: mass chromatogram, middle: mass spectrum of UV/Vis peak, bottom: UV/Vis chromatogram).

NMR data

Overlay of screening sample (compound 6) and resynthesized compound 6-1

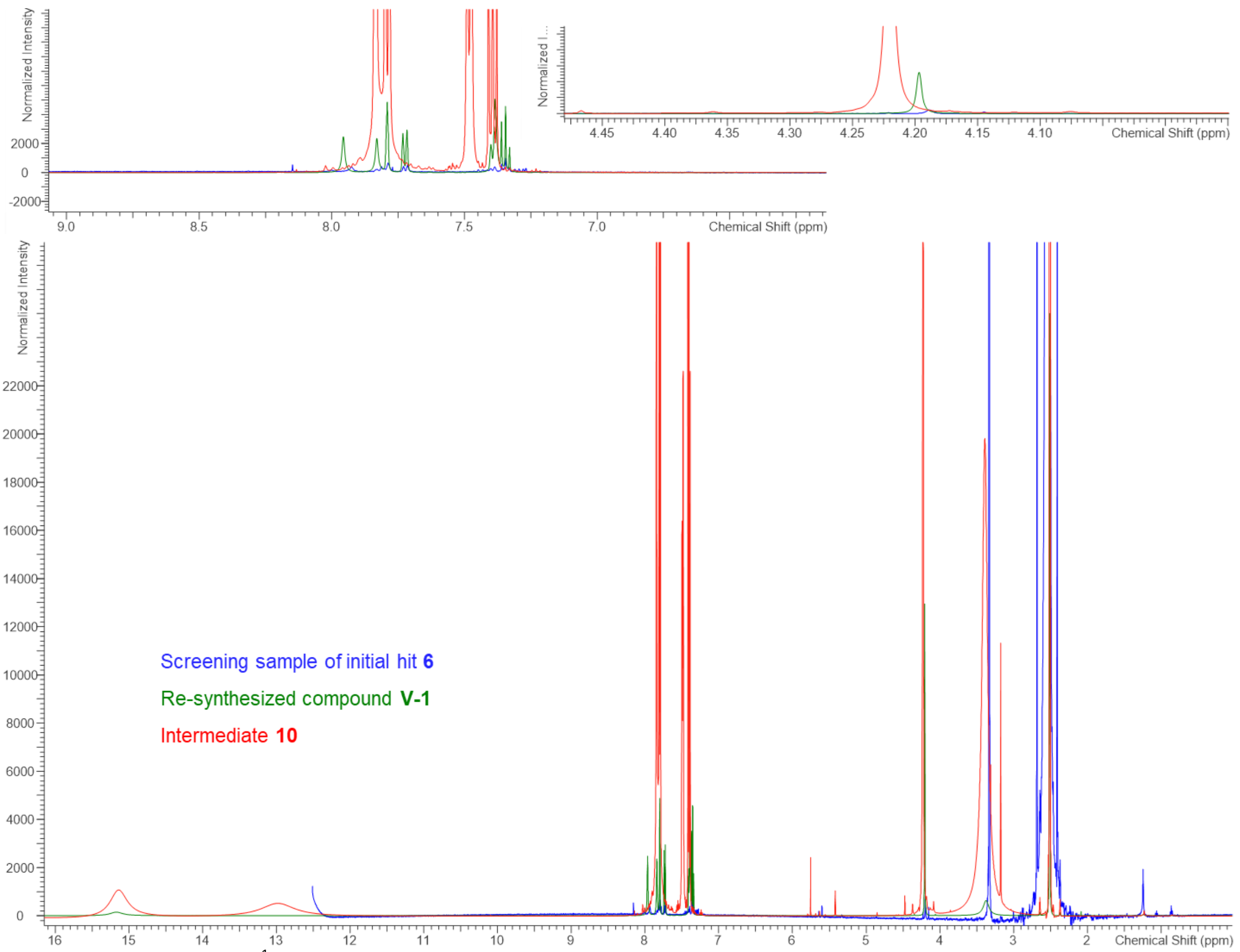

Figure S4: Comparison of ^{1}H NMR spectra (500 MHz) of screening sample (hit **6**) and resynthesized compound **6-1** with crucial

zoom regions of aromatic and aliphatic regions. The screening sample was measured in HDMSO, whereas the other sample was dissolved in DMSO-d_6.

*3-(((1*H*-1,2,3-Triazol-5-yl)thio)methyl)benzoic acid (10)*

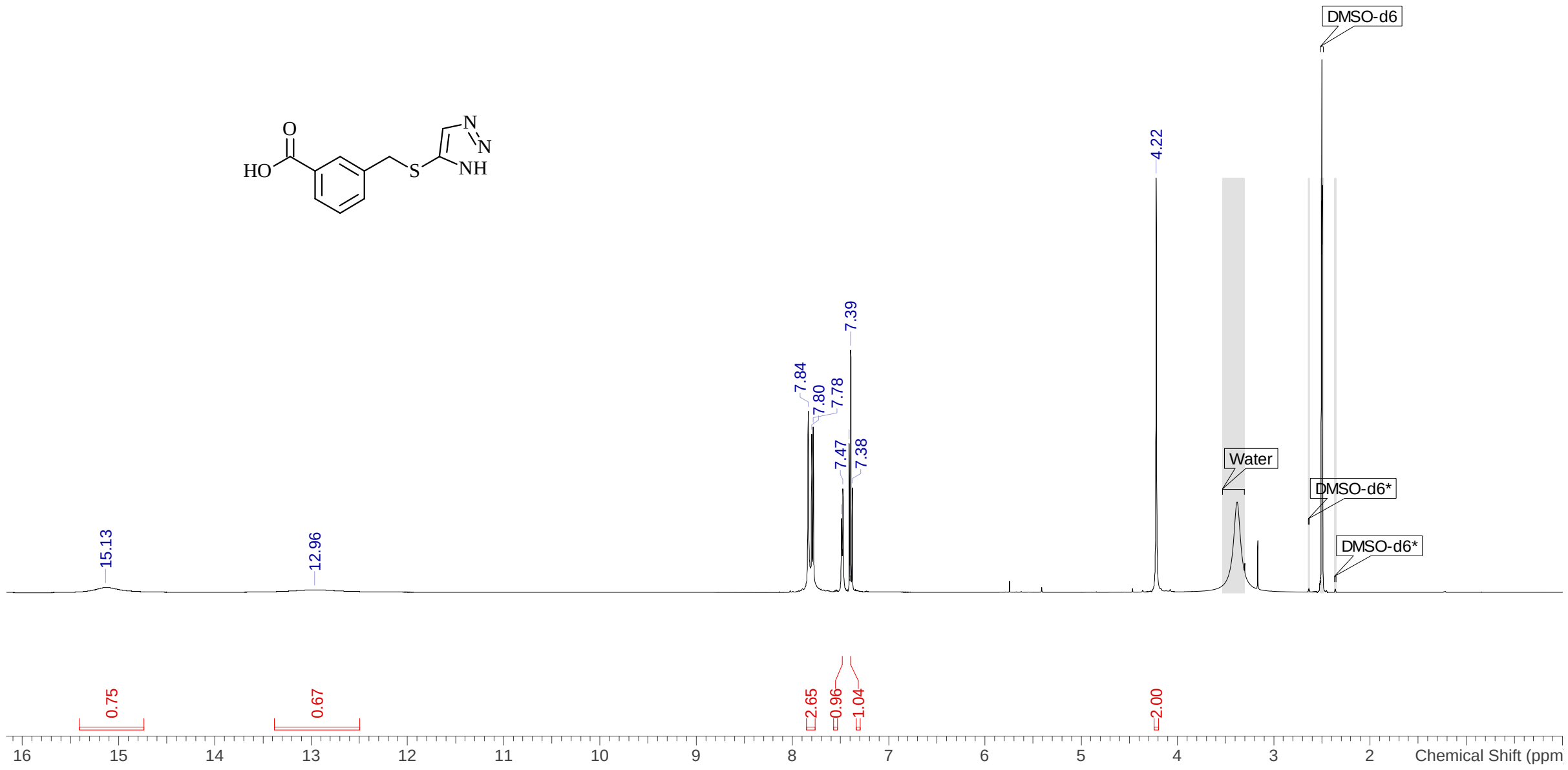

Figure S5: ^{1}H NMR spectrum of **10** measured in DMSO-d_6 at 500 MHz.

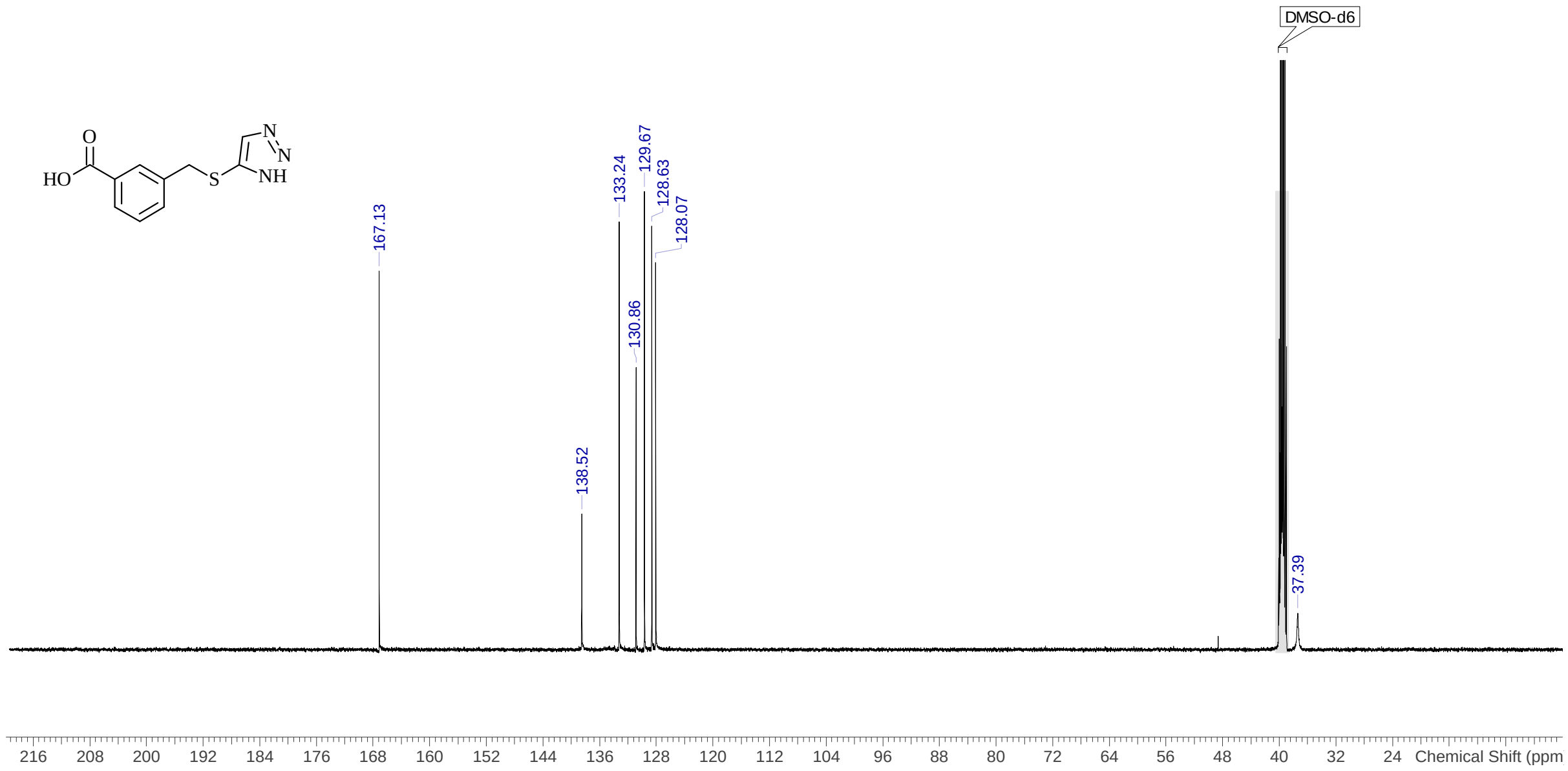

Figure S6: ^{13}C NMR spectrum of **10** measured in DMSO-d_6 at 126 MHz.

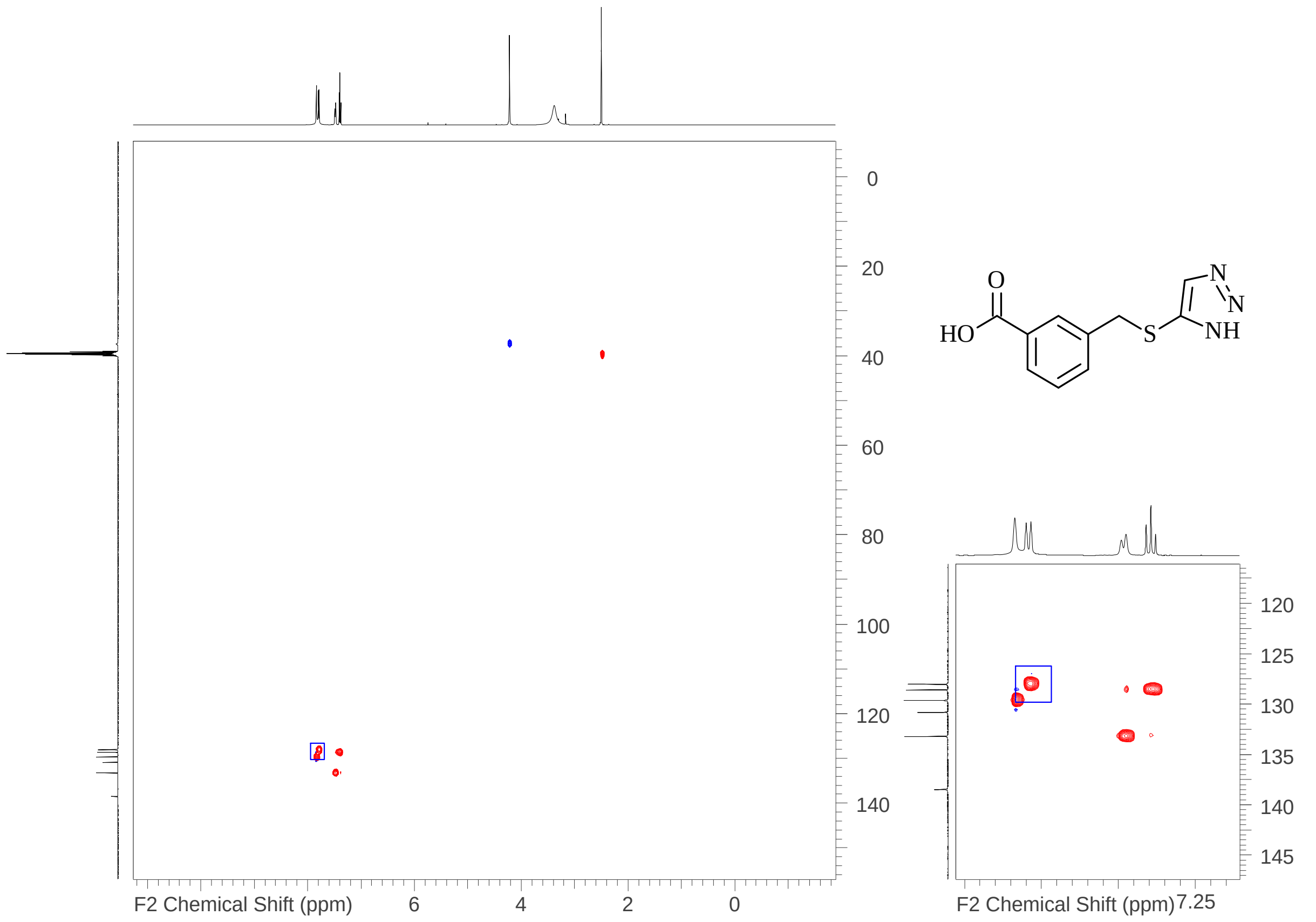

Figure S7: HSQC spectrum of **10** measured in DMSO-d_6 at 500/126 MHz; marked signals (blue box) are the overlapping ones in ^{13}C NMR spectrum.

*3-(((1*H*-1,2,3-Triazol-5-yl)sulfinyl)methyl)benzoic acid (6-1)*

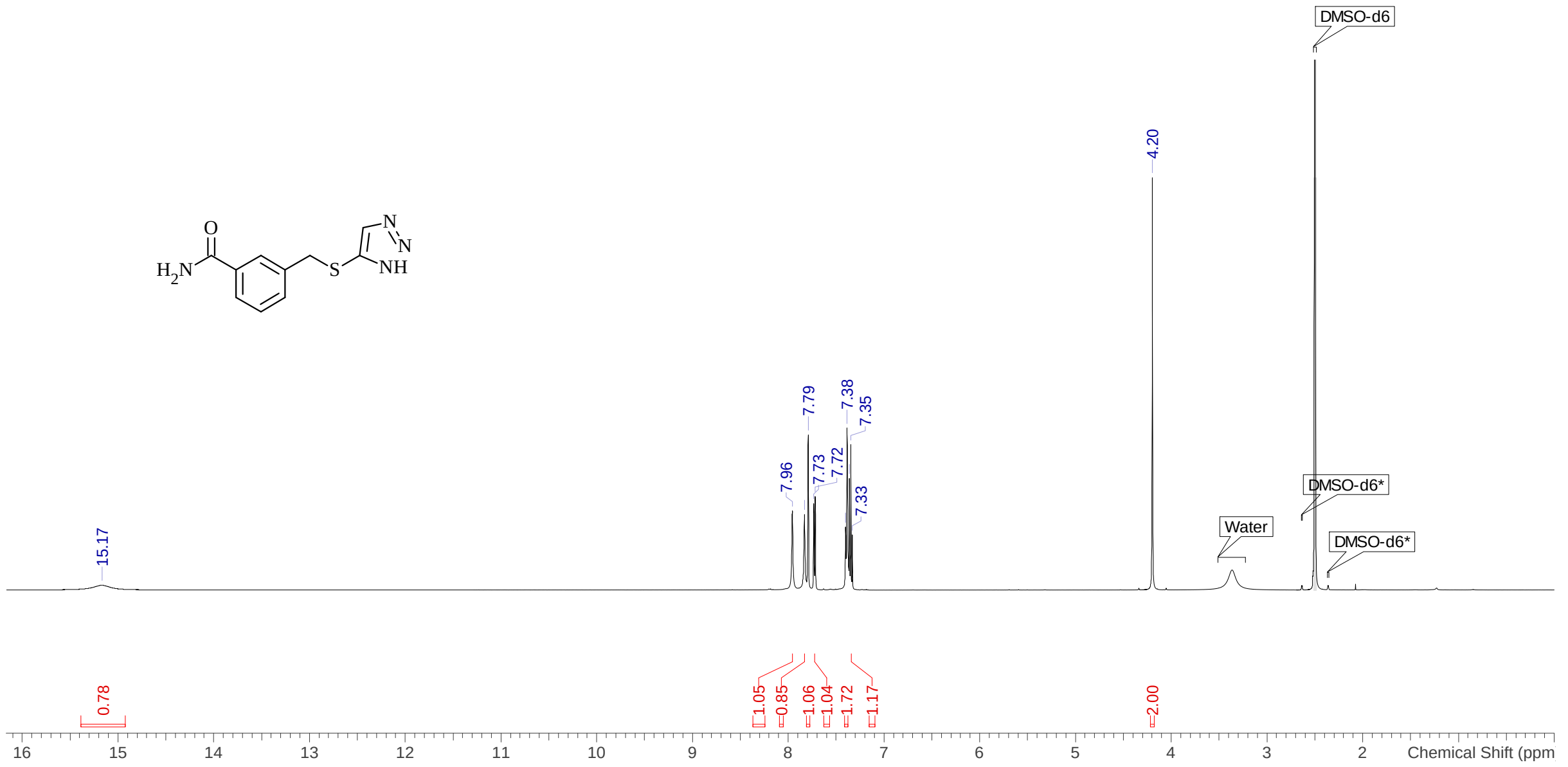

Figure S8: ^{1}H NMR spectrum of **6-1** measured in DMSO-d_6 at 500 MHz.

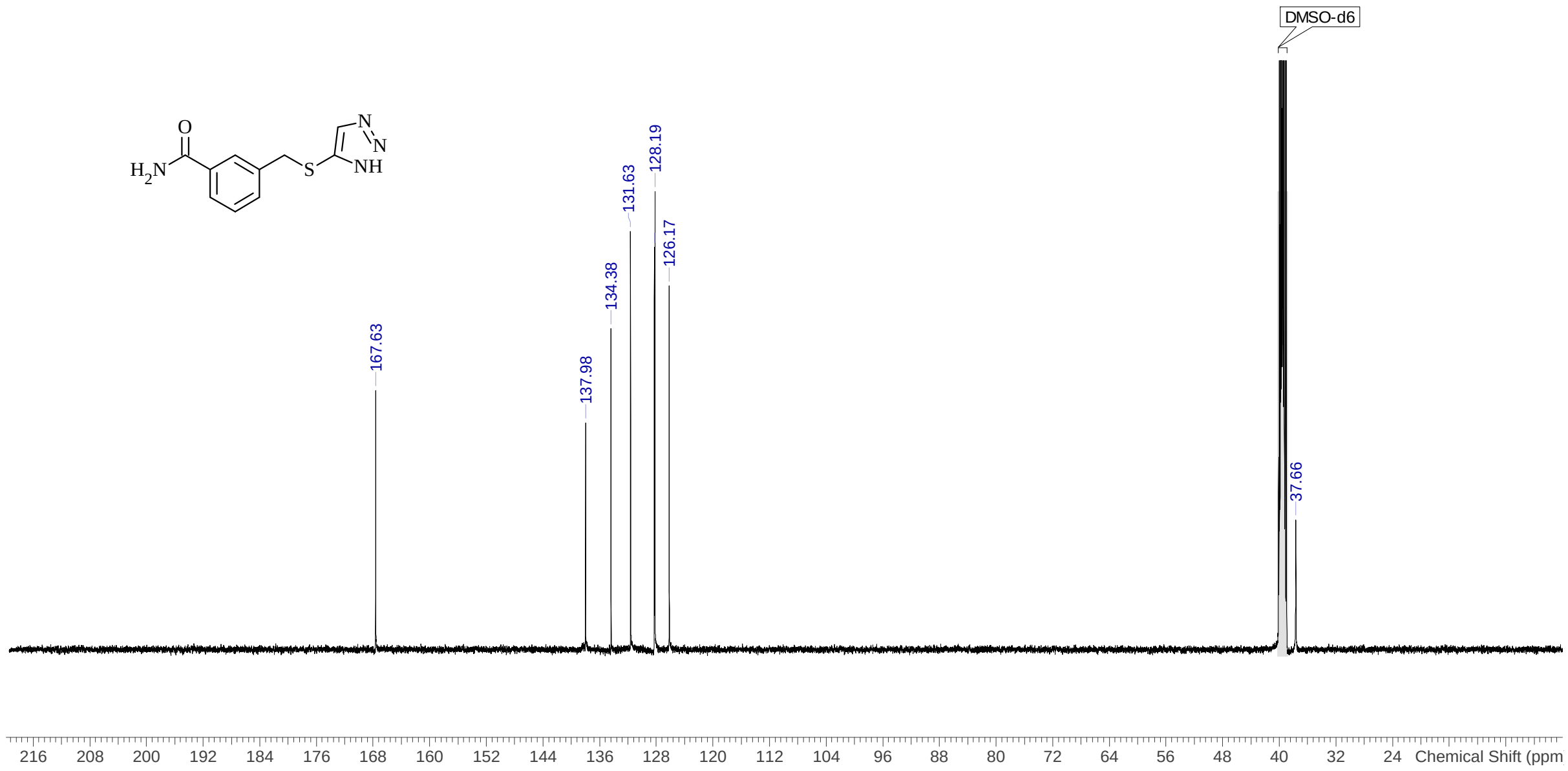

Figure S9: ^{13}C NMR spectrum of **6-1** measured in DMSO-d_6 at 126 MHz.

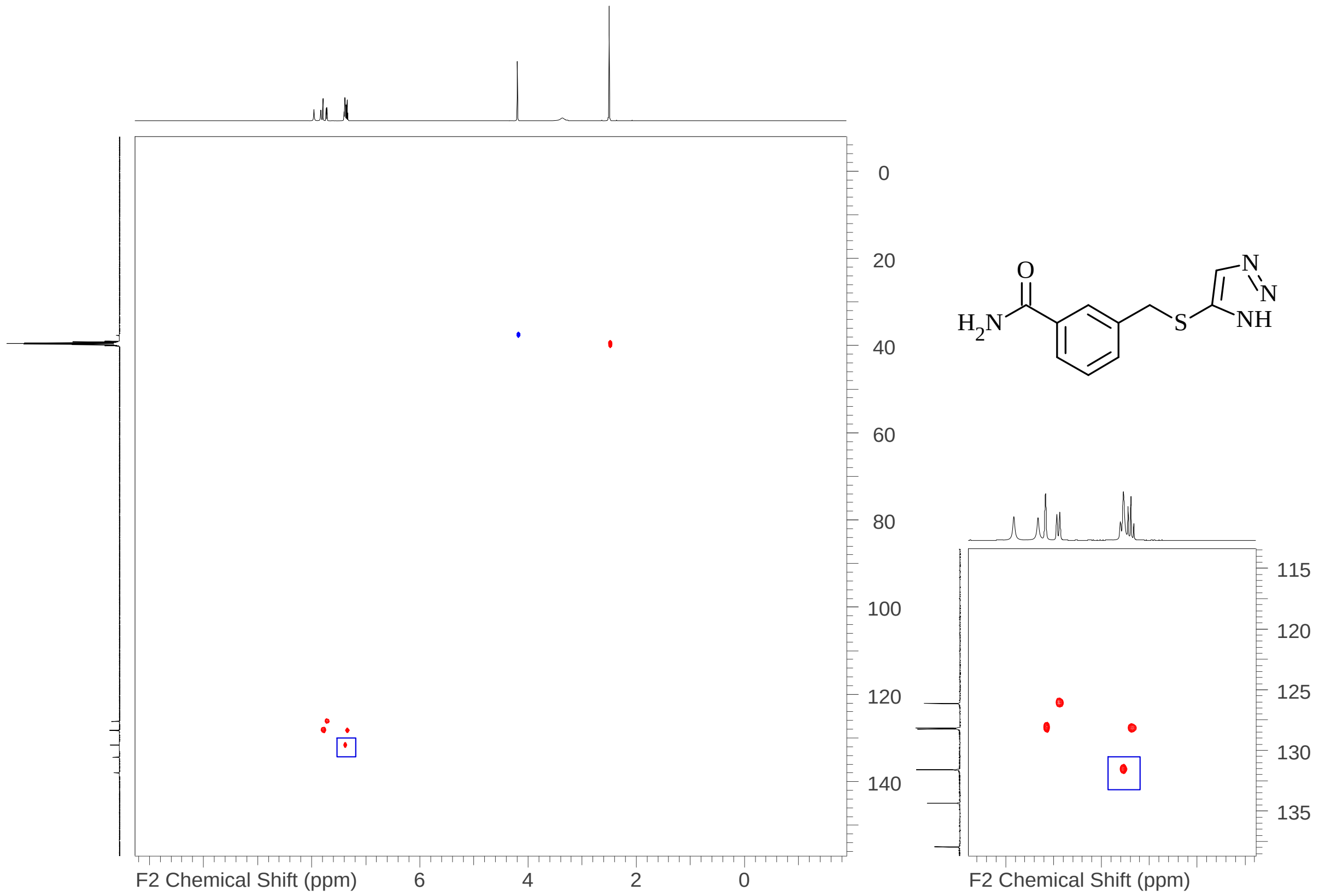

Figure S10: HSQC spectrum of **6-1** measured in DMSO-d_6 at 500/126 MHz; marked signals (blue box) are the overlapping ones in ^{13}C NMR spectrum.

HRMS data

*3-(((1*H*-1,2,3-Triazol-5-yl)sulfinyl)methyl)benzoic acid (6-1)*

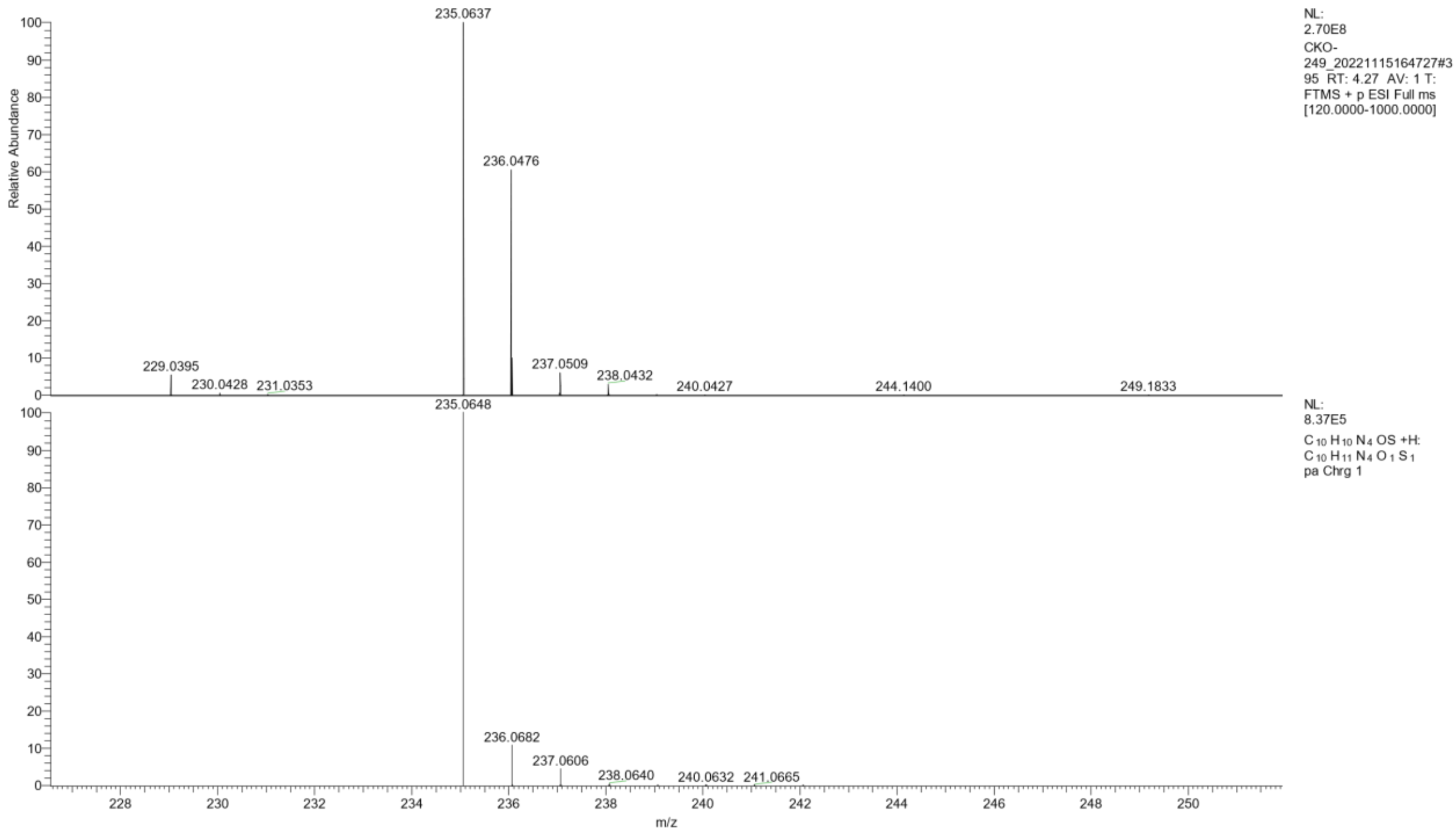

Figure S11: HRMS spectra of **6-1** (top: measured spectrum, bottom: calculated spectrum).

Dose-response curves

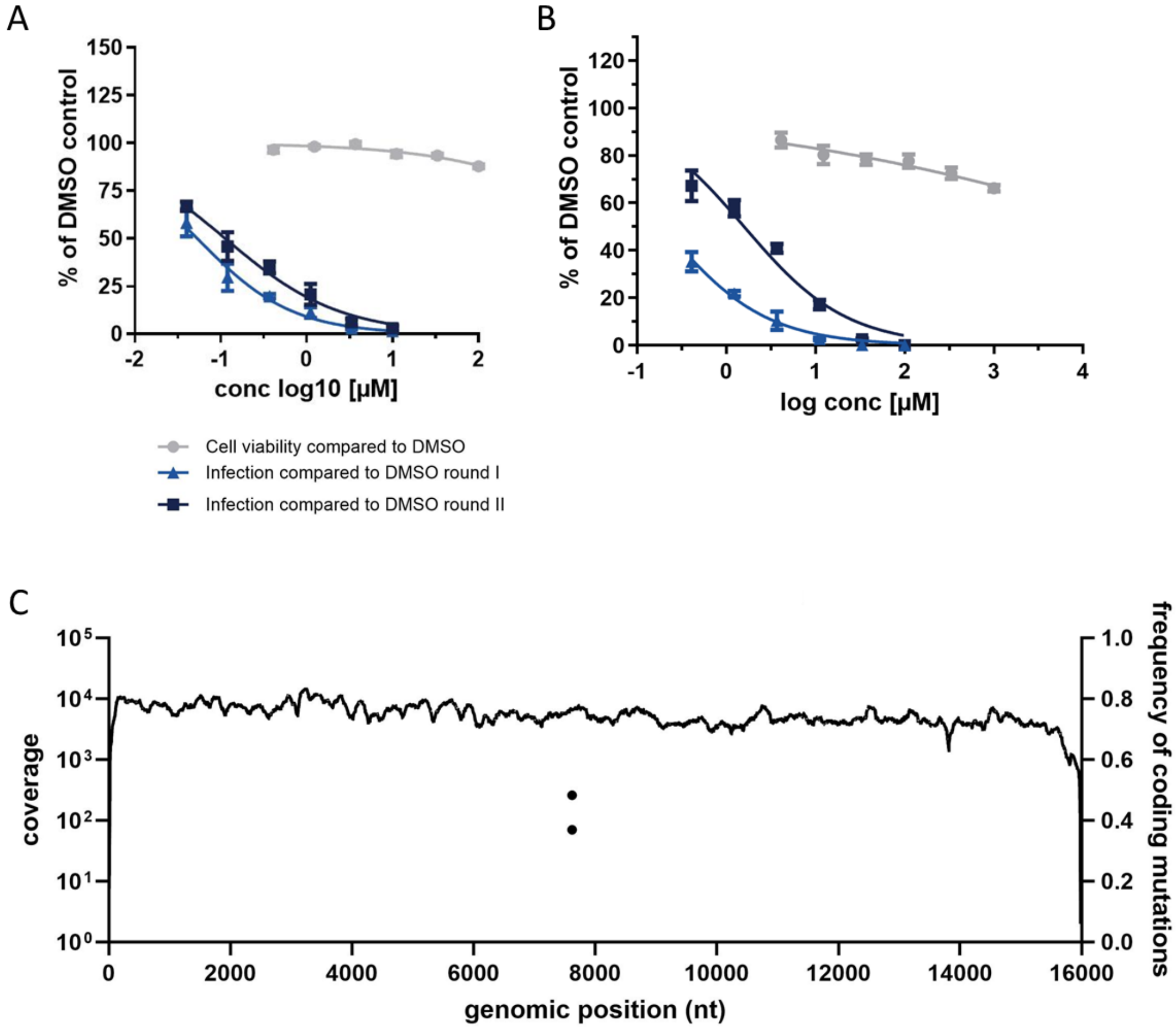

Figure S12: (A-B) Dose-response curves for RSV infection and HEp-2 cell viability. X-axis represents % residual RSV infection as determined by luciferase reporter signal measurements. (A) initial HTS hit **6** (B) ribavirin (C) Coverage plot of adapted virus population.

Compound class 7

LC-MS data

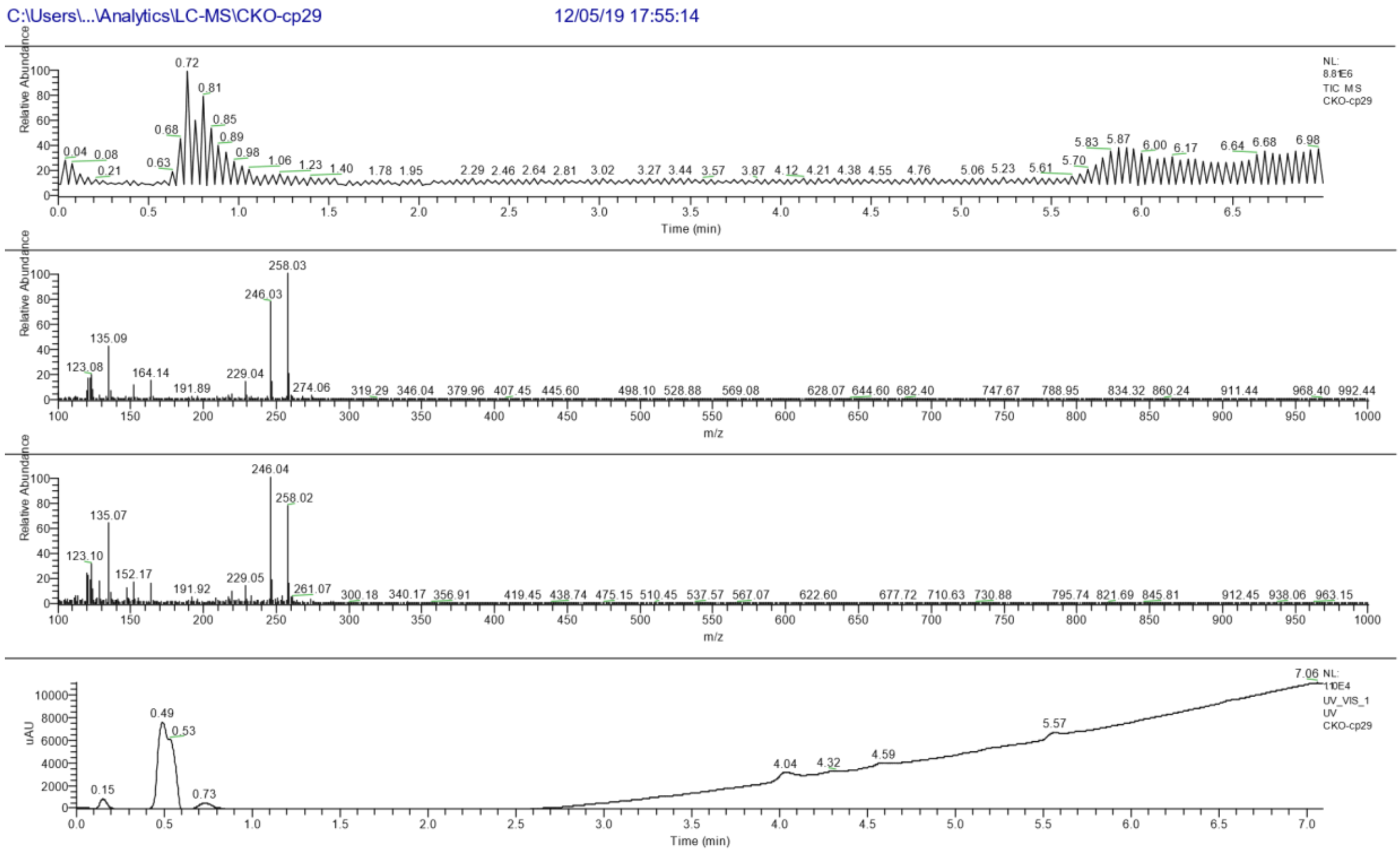

Figure S13: LC-MS data of initial screening sample (compound **7a**); top: mass chromatogram, middle: mass spectra of the three UV peaks, bottom: UV chromatogram.

*1-(1-Methyl-1*H*-imidazol-2-yl)*-N-*((5,6,7,8-tetrahydroimidazo[1,2-a]pyridin-8-yl)methyl)methanamine (7a-1)*

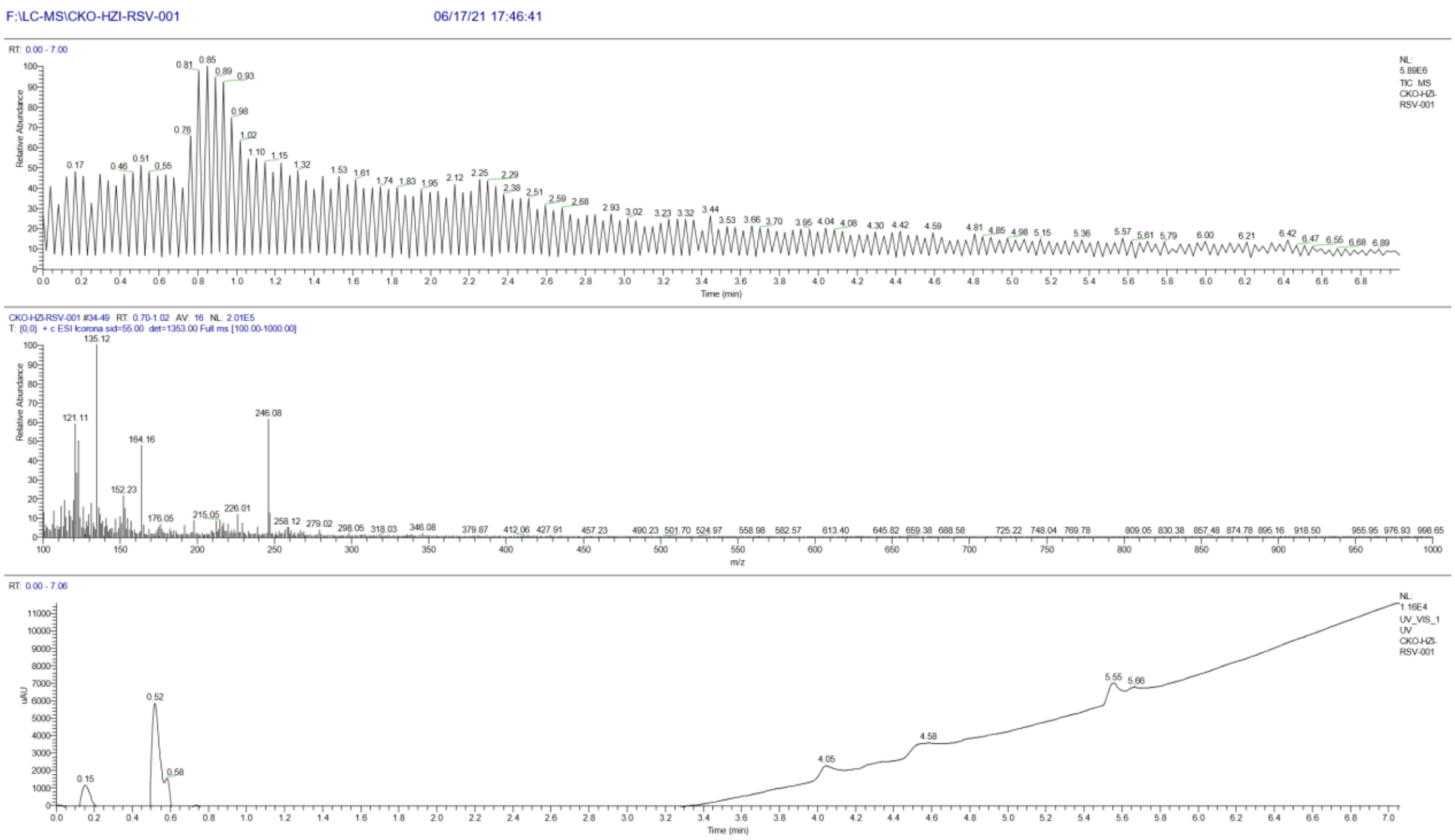

Figure S14: LC-MS data of **7a-1**; top: mass chromatogram, middle: mass spectra of the three UV peaks, bottom: UV chromatogram.

NMR data

Overlay of screening sample (compound 7) and resynthesized compound 7-1

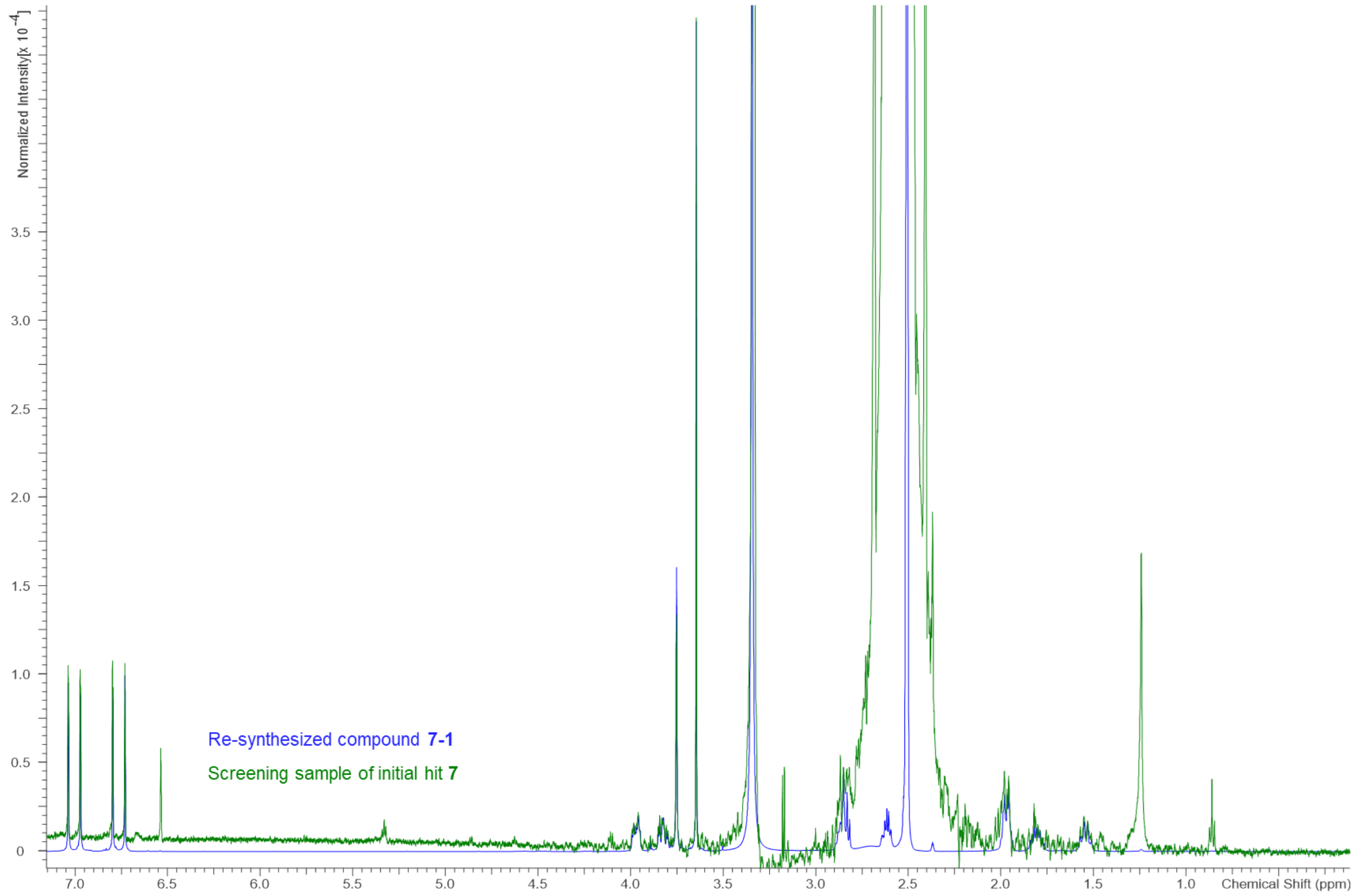

Figure S15: Comparison of ^{1}H NMR spectra (500 MHz) of screening sample (hit **7**) and resynthesized compound **7-1**. The screening sample was measured in HDMSO, whereas the other sample was dissolved in DMSO-d_6.

*1-(1-Methyl-1*H*-imidazol-2-yl)*-N-*((5,6,7,8-tetrahydroimidazo[1,2-a]pyridin-8-yl)methyl)methanamine (7a-1)*

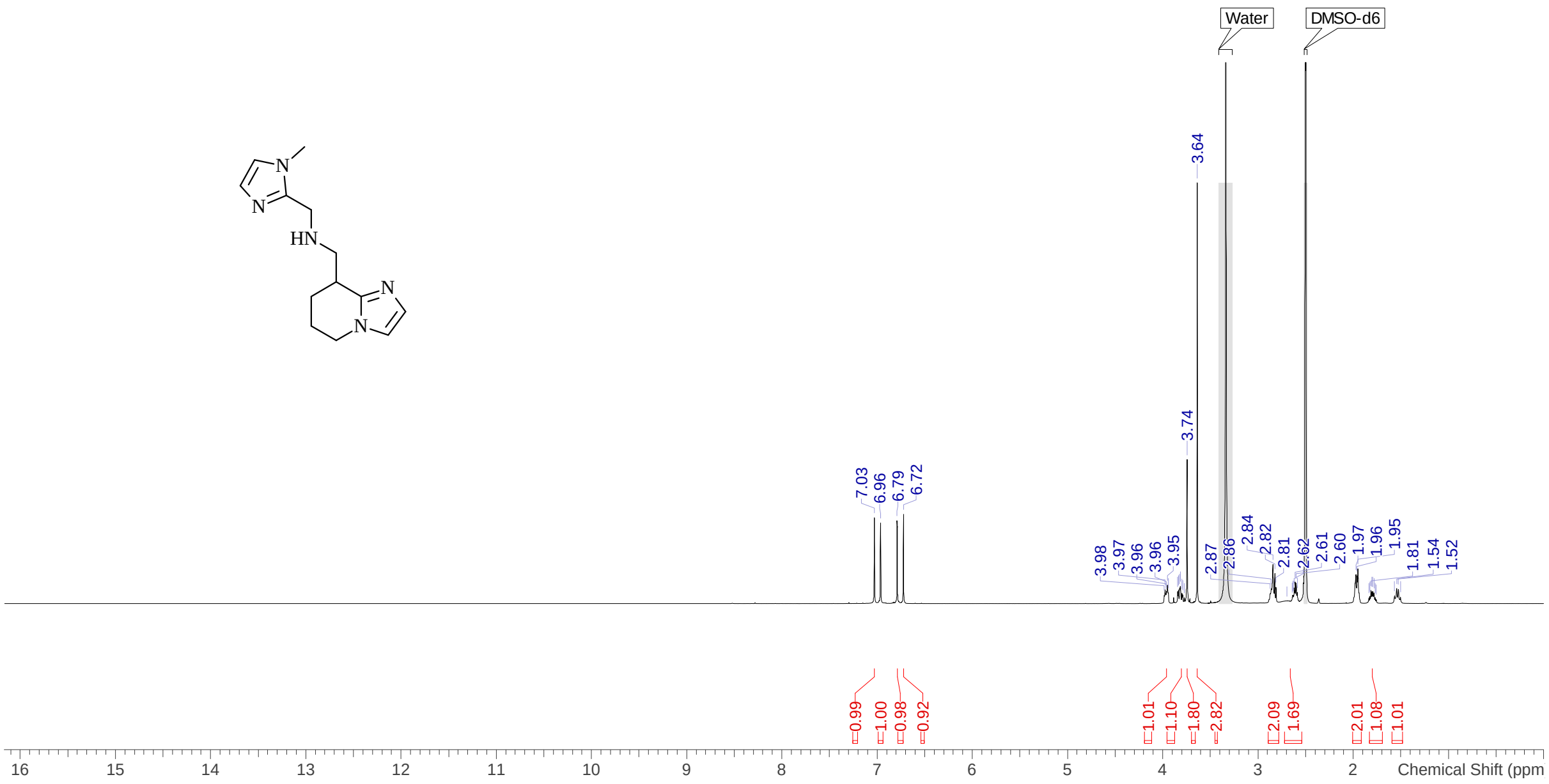

Figure S16: ^{1}H NMR spectrum of **7a-1** measured in DMSO-d_6 at 500 MHz.

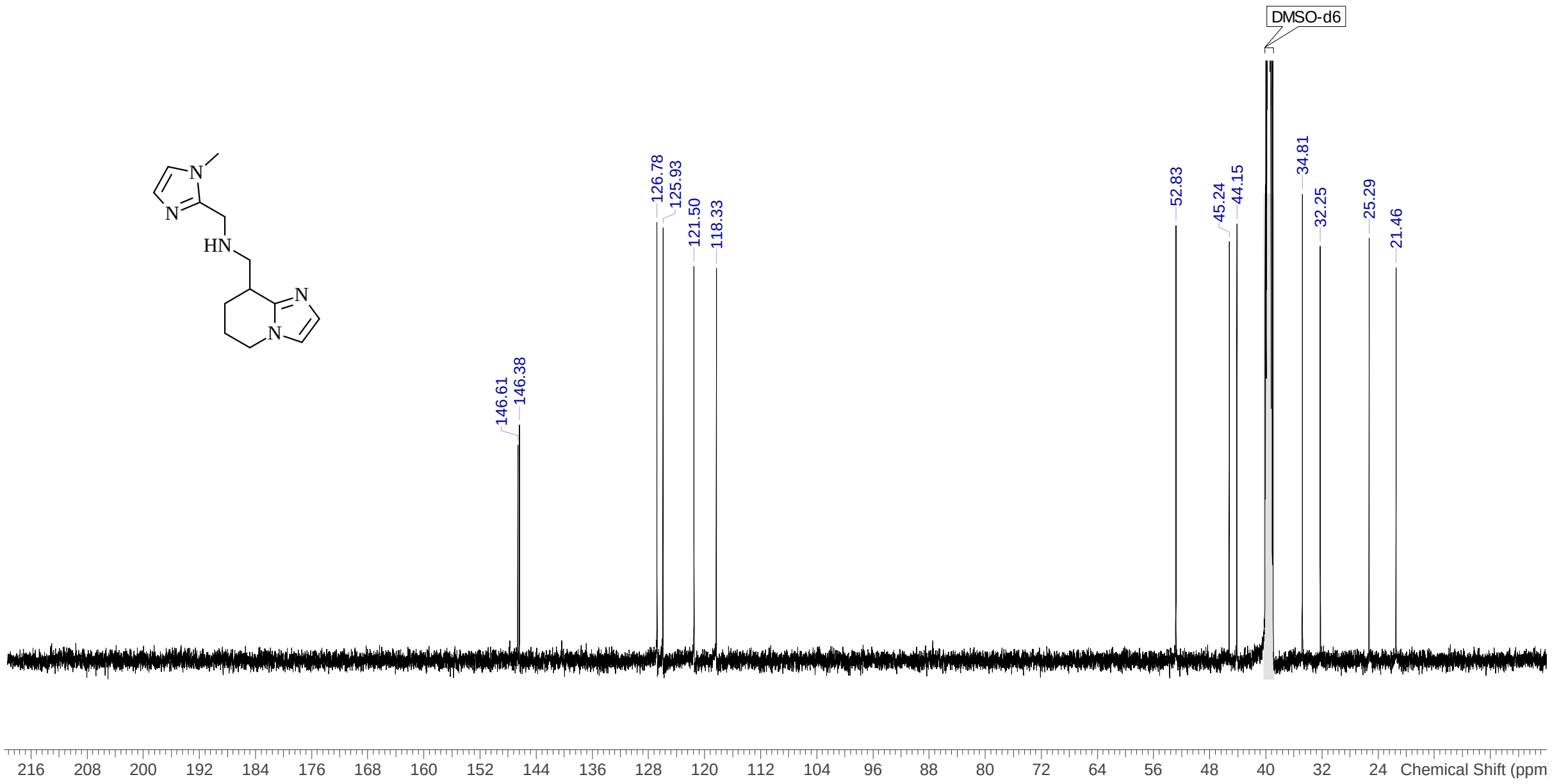

Figure S17: ^{13}C NMR spectrum of **7a-1** measured in DMSO-d_6 at 126 MHz.

HRMS data

*1-(1-Methyl-1*H*-imidazol-2-yl)*-N-*((5,6,7,8-tetrahydroimidazo[1,2-a]pyridin-8-yl)methyl)methanamine (7a-1)*

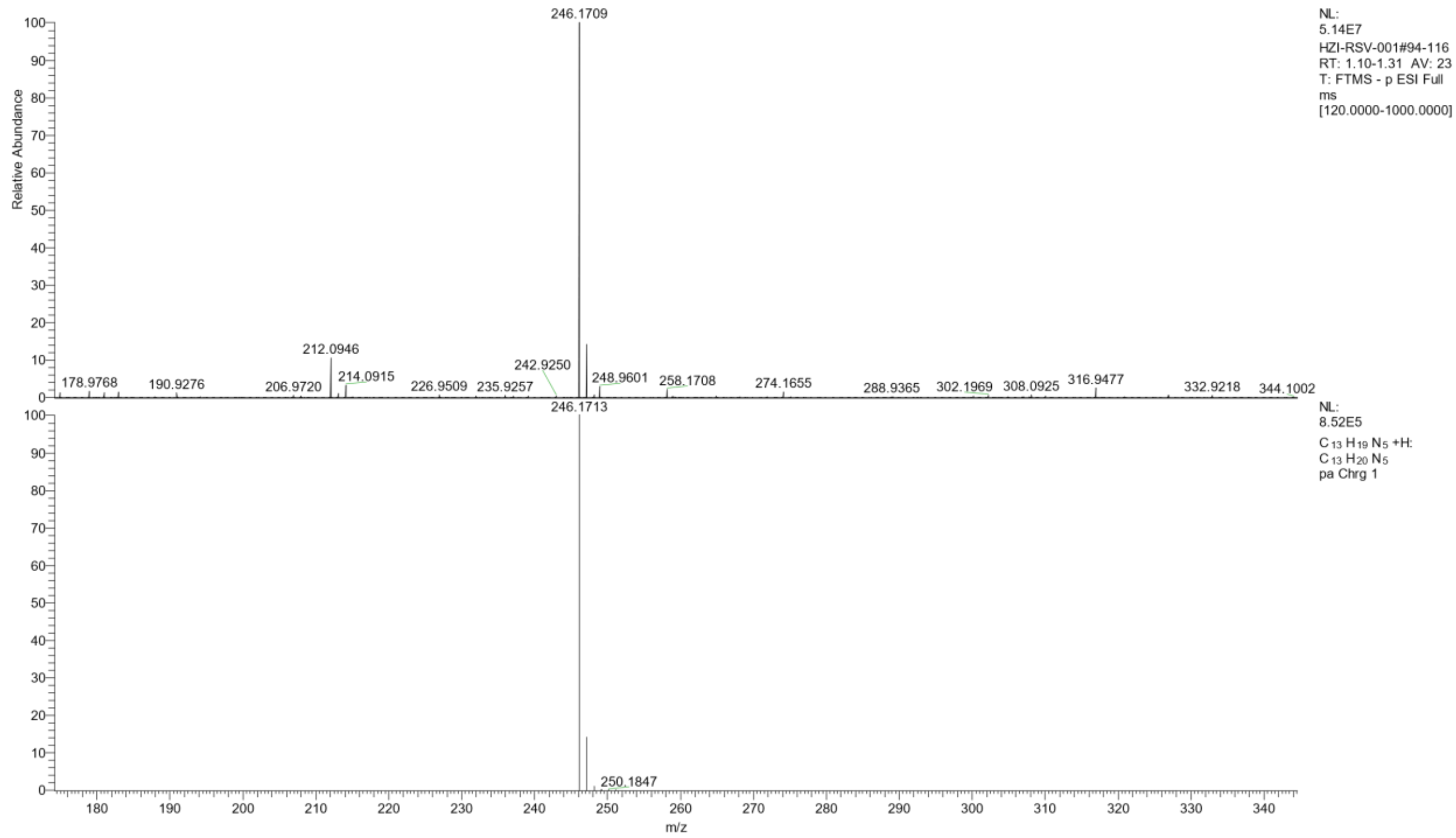

Figure S18: HRMS spectra of **7a-1** (top: measured spectrum, bottom: calculated spectrum).

Dose-response curves

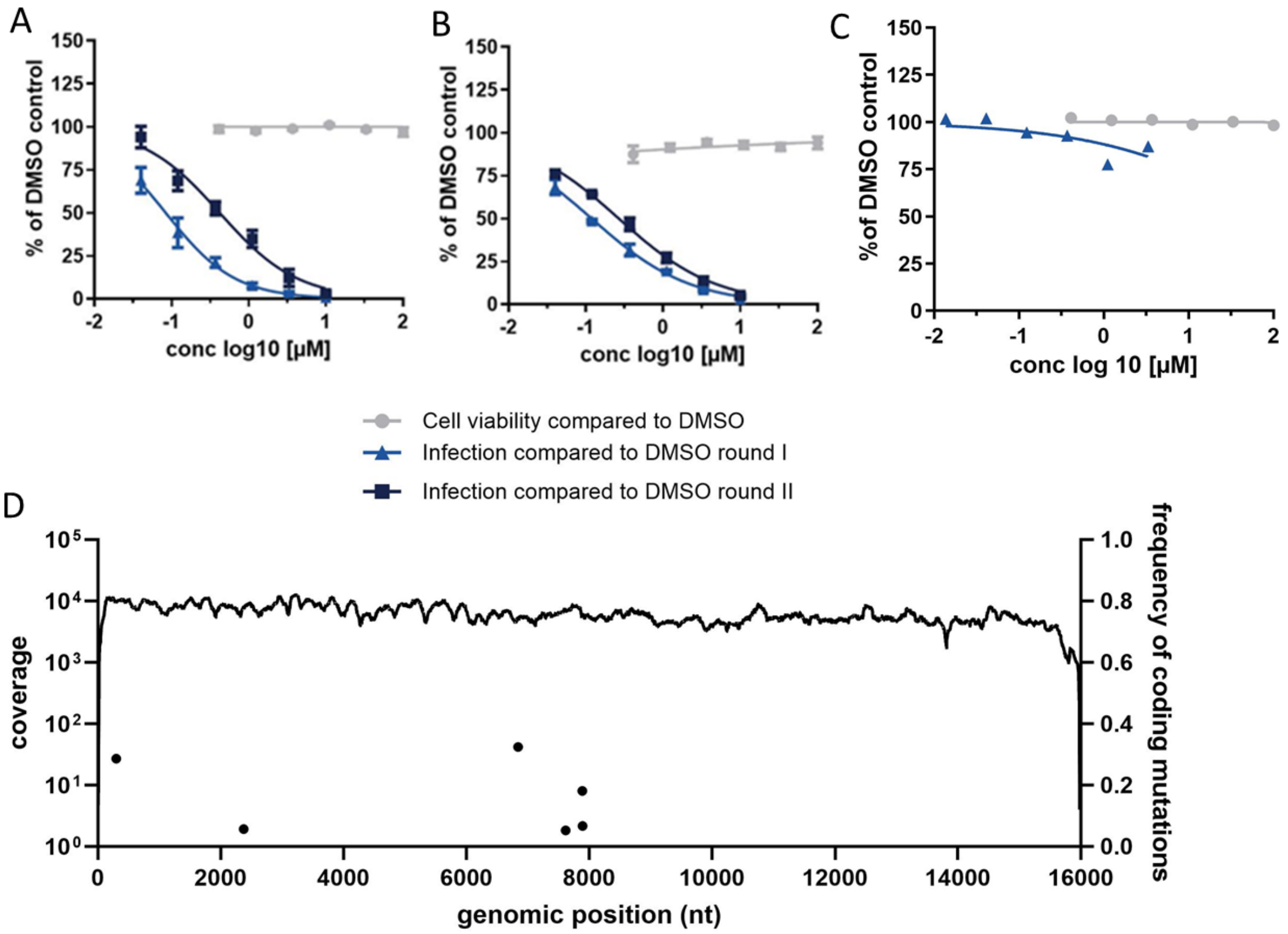

Figure S19: Dose-response curves of initial HTS hits **7a** (A, counts of experiments (n) = 3), **7b** (B, n= 3), and **7a-1** (C, n = 1) for RSV infection and HEp-2 cell viability. X-axis represents % residual RSV infection as determined by luciferase reporter signal measurements. (D) Coverage plot for adapted virus population with compound **7a**.
